# Podoplanin Antibody SZ168 Alleviates Sepsis Inflammation and Macrophage Dysregulation via ERK Signaling

**DOI:** 10.1155/humu/3791421

**Published:** 2026-06-08

**Authors:** Hongbin Wang, Junfeng Heng, Yuhong Zhang, Jie Cui, Yanyan Li, Sun Yu, Xiaolei Gu, Shiqi Lu, Yiming Zhao

**Affiliations:** ^1^ Department of Emergency, The First Affiliated Hospital of Soochow University, Suzhou, Jiangsu Province, China, sdfyy.cn; ^2^ Department of Emergency, Jiangyin Hospital Affiliated to Nanjing University of Chinese Medicine, Jiangyin, Jiangsu Province, China, njucm.edu.cn; ^3^ Intensive Care Unit, Wuxi People′s Hospital, Wuxi, Jiangsu Province, China; ^4^ Department of Oncology, Affiliated Jiangyin Hospital of Nantong University, Jiangyin, Jiangsu Province, China; ^5^ Department of Clinical Laboratory, Changning Maternity and Infant Health Hospital, East China Normal University, Shanghai, China, ecnu.edu.cn; ^6^ Department of Emergency, Minhang Hospital, Fudan University, Shanghai, China, fudan.edu.cn; ^7^ Department of Emergency, Changshu Hospital Affiliated to Soochow University (First People′s Hospital of Changshu City), Changshu, Jiangsu Province, China; ^8^ Department of Emergency, Affiliated Changshu Hospital of Nantong University, Changshu, Jiangsu Province, China; ^9^ Jiangsu Institute of Hematology, The First Affiliated Hospital of Soochow University, Suzhou, Jiangsu Province, China, sdfyy.cn

**Keywords:** macrophage, monoclonal antibody, podoplanin, polarization, sepsis

## Abstract

**Background:**

Sepsis is a life‐threatening condition marked by excessive inflammation and immune dysregulation. Macrophages are central to this process. Podoplanin (PDPN), a transmembrane glycoprotein, is upregulated in inflammatory macrophages, but its role in sepsis remains unclear.

**Objective:**

The aim of this study is to investigate the role of PDPN and the therapeutic potential of its monoclonal antibody SZ168 in LPS‐induced macrophages and to extend these observations with supplementary in vivo and preliminary clinical validation.

**Methods:**

RAW264.7 macrophages were stimulated with LPS and treated with SZ168. PDPN expression and the effects of SZ168 on cytokine secretion, polarization, apoptosis, and ERK signaling were assessed by proteomics, qPCR, Western blotting, ELISA, and flow cytometry. siRNA‐mediated PDPN knockdown was used to validate pathway dependence. During revision, additional Western blot validation of MEK, p90RSK, and c‐Fos; supplementary in vivo experiments in a mouse sepsis model; and a preliminary clinical cohort analysis were incorporated to strengthen mechanistic and translational relevance.

**Results:**

LPS stimulation significantly upregulated PDPN expression at both mRNA and protein levels in macrophages. SZ168 treatment reduced LPS‐induced secretion of IL‐6, TNF‐*α*, and IL‐1*β*; promoted anti‐inflammatory polarization; and attenuated apoptosis. Revision‐stage Western blot analyses further showed coordinated recovery of MEK, p90RSK, c‐Fos, and p‐ERK together with suppression of SERPINE1 after SZ168 treatment, especially in PDPN‐knockdown cells. Supplementary in vivo experiments showed lower BUN, creatinine, inflammatory cytokines, and lung injury after SZ168 treatment. In a preliminary clinical cohort, serum PDPN levels were higher in patients with sepsis than in controls (23.81 ± 17.64 vs. 3.53 ± 0.58, *p* < 0.001).

**Conclusion:**

The PDPN monoclonal antibody SZ168 attenuates inflammatory responses, promotes anti‐inflammatory macrophage polarization, and inhibits apoptosis through modulation of the ERK signaling pathway in LPS‐induced macrophages. The supplementary in vivo and preliminary human data added during revision further support the translational relevance of PDPN as a therapeutic target in sepsis.

## 1. Introduction

Sepsis is a life‐threatening condition characterized by a dysregulated host immune response to infection, resulting in widespread inflammation and multiple organ dysfunction—including damage to the heart, liver, lungs, and kidneys [1]. Despite heightened clinical awareness and advances in supportive care, sepsis remains a major global health concern, with persistently high incidence and mortality rates [2]. The pathophysiology of sepsis is highly complex and involves an uncontrolled inflammatory cascade marked by a cytokine storm, immune system dysfunction, and a breakdown in immune homeostasis [1, 3]. Current therapeutic approaches largely focus on anti‐inflammatory agents, antimicrobial therapy, and strategies to restore immune balance [3, 4]. Emerging evidence has highlighted the central roles of various immune effector cells—including macrophages, dendritic cells, and natural killer cells—in orchestrating the immune dysregulation seen in sepsis [5–7].

Macrophages are essential components of the innate immune system, functioning to maintain immune equilibrium and defend against pathogens through recognition, phagocytosis, and elimination of invading microorganisms [8]. Known for their remarkable heterogeneity and plasticity, macrophages can respond to a wide range of microenvironmental stimuli [8]. This adaptability enables them to polarize into distinct phenotypes, most notably the proinflammatory M1 and anti‐inflammatory M2 subtypes [9]. Stimulation with lipopolysaccharide (LPS) and interferon‐*γ* (IFN‐*γ*) promotes M1 polarization, while cytokines such as interleukin‐4 (IL‐4) and interleukin‐10 (IL‐10) drive M2 polarization [9]. Under normal physiological conditions, a dynamic balance exists between M1 and M2 macrophages. However, during infection or tissue injury, this equilibrium is disrupted as macrophages initiate inflammatory responses and exert phenotype‐specific functions depending on the stage and context of disease progression [10]. Macrophage polarization is tightly regulated by a complex network of factors, including transcriptional and epigenetic regulators, cellular senescence and death pathways, hypoxia, gut microbiota, and metabolic intermediates [11]. Recent research has increasingly underscored the pivotal role of macrophages in regulating the immune response and maintaining immune homeostasis during sepsis, positioning them as promising therapeutic targets.

Podoplanin (PDPN) is a highly conserved mucin‐type transmembrane glycoprotein and the only known endogenous ligand for C‐type lectin‐like receptor 2 (CLEC‐2). PDPN′s primary physiological function involves promoting platelet activation and aggregation via its interaction with CLEC‐2, which is abundantly expressed on the surface of platelets [12]. While PDPN is typically expressed in nonhematopoietic cells such as fibroblasts and epithelial cells [12], it is minimally expressed in resting macrophages. However, PDPN expression is markedly upregulated in macrophages upon LPS stimulation, particularly in pathological settings involving inflammation or cancer [13, 14]. In sepsis, the PDPN–CLEC‐2 interaction plays a crucial role in immune regulation by facilitating macrophage recruitment to sites of infection and enhancing bacterial clearance [15]. Moreover, in systemic inflammatory conditions such as zymosan‐induced sepsis, splenic PDPN^+^ macrophages exhibit increased phagocytic activity and expansion in number, underscoring their functional relevance [16]. Nonetheless, the specific role of PDPN in regulating macrophage function and polarization during sepsis remains largely unclear.

In this study, we first established an LPS‐stimulated macrophage model to evaluate changes in PDPN expression. We then employed the monoclonal antibody SZ168, an agent known to interfere with PDPN‐dependent signaling, to assess its effects on macrophage proliferation, apoptosis, and polarization. Proteomic analysis and revision‐stage pathway extension experiments were performed to elucidate the molecular mechanisms underlying PDPN function in LPS‐stimulated macrophages. A PDPN knockdown model using small interfering RNA (siRNA) (si‐PDPN) was developed to further validate the role of PDPN in LPS‐induced macrophage responses. To improve translational relevance during revision, we also incorporated supplementary in vivo mouse data and a preliminary clinical cohort analysis. Collectively, these findings advance the understanding of PDPN′s role in sepsis and provide additional support for its therapeutic potential.

## 2. Methods

### 2.1. Cell Culture and Stimulation

The murine macrophage cell line RAW264.7 was used for all in vitro experiments. Cells were obtained from the Cell Resource Center of the Chinese Academy of Sciences (CTCC, HCL‐0102, China) and maintained in high‐glucose Dulbecco′s Modified Eagle′s Medium (DMEM; SH30243.01, HyClone, United States), supplemented with 10% fetal bovine serum (FBS; A511‐001, Lonsera, Uruguay) and 1% penicillin–streptomycin (SV30010, Biosharp, China). Cells were cultured at 37°C in a humidified atmosphere containing 5% CO_2_.

To establish an in vitro sepsis‐like macrophage model, RAW264.7 cells were stimulated with LPS (10 ng/mL, L8880, Solarbio, China) for 24 h to induce proinflammatory cytokine secretion and polarization toward the M1 phenotype.

To evaluate the cytotoxicity of the monoclonal anti‐PDPN antibody SZ168, RAW264.7 cells were divided into seven treatment groups: control (0 *μ*g/mL) and SZ168 at concentrations of 0.625, 1.25, 2.5, 5, 10, and 20 *μ*g/mL. Cell viability was assessed to determine the optimal nontoxic working concentration.

To investigate the immunomodulatory role of SZ168 in LPS‐induced macrophages, cells were divided into four groups: control, LPS, LPS + SZ168 (2.5 *μ*g/mL), and LPS + SZ168 (5 *μ*g/mL).

To further explore the molecular mechanisms of PDPN function, RAW264.7 cells were transfected with siRNA targeting PDPN. Cells were divided into six experimental groups: control, LPS, LPS + SZ168 (5 *μ*g/mL), LPS + negative control siRNA (siRNA‐NC), LPS + PDPN‐specific siRNA (siRNA‐PDPN), and LPS + siRNA‐PDPN + SZ168. These experimental setups were used to assess the effects of PDPN knockdown and SZ168 treatment on macrophage function under inflammatory conditions.

#### 2.1.1. Supplementary In Vivo Validation

To provide additional in vivo evidence during revision, we analyzed a supplementary mouse sepsis dataset comprising control, model, model + SZ168 (50 *μ*g/kg), and model + SZ168 (100 *μ*g/kg) groups. Renal injury markers (BUN and creatinine), inflammatory cytokines in organ supernatants and serum (IL‐1*β*, IL‐6, and TNF‐*α*), lung histopathology by hematoxylin–eosin staining, and immunofluorescence staining for F4/80 with CD86 or CD206 were evaluated and summarized as Supporting Information 1: Figure S3.

### 2.2. Quantification of PDPN mRNA Expression

Total RNA was extracted from RAW264.7 macrophage cells using a commercial RNA extraction kit (M004, Puhe, Wuxi, China) according to the manufacturer′s protocol. Complementary DNA (cDNA) was synthesized from 1 *μ*g of total RNA using a reverse transcription kit (PC1703, Aidlab, Beijing, China). Quantitative real‐time PCR (qRT‐PCR) was performed using a SYBR Green‐based premix (PC3302, Aidlab, Beijing, China) and a real‐time PCR detection system (ABI 7500, United States). The following primers were used for PDPN: forward: 5 ^′^‐ACTGTCCACCTCAGCAAC‐3 ^′^, reverse: 5 ^′^‐ACGCCAACTATGATTCCA‐3 ^′^. GAPDH was used as the internal reference gene, with the following primer sequences: forward: 5 ^′^‐GGTGAAGGTCGGTGTGAACG‐3 ^′^, reverse: 5 ^′^‐CTCGCTCCTGGAAGATGGTG‐3 ^′^. PCR cycling conditions were as follows: initial denaturation at 95°C for 10 min, followed by 40 cycles of denaturation at 95°C for 20 s, annealing at 55°C for 20 s, and extension at 72°C for 20 s. A final dissociation stage at 95°C for 15 s was performed to verify specificity. Relative mRNA expression levels were calculated using the 2^−*Δ* *Δ* *C* *t*^ method and normalized to GAPDH expression.

### 2.3. Flow Cytometry

Flow cytometry was used to assess macrophage polarization and apoptosis in the LPS‐treated cell model.

To evaluate macrophage polarization, surface markers CD86 (M1 phenotype) and CD206 (M2 phenotype) were analyzed. Briefly, RAW264.7 cells were harvested using 0.25% trypsin (002PI, CTCC, China) for 10 min at room temperature, followed by centrifugation at 1200 rpm for 15 min. Cell pellets were washed three times with phosphate‐buffered saline (PBS) and resuspended at a concentration of 1 × 10^5^ cells/mL. Fluorescently labeled anti‐CD86 and anti‐CD206 antibodies were added to the cell suspension and incubated for 15 min at room temperature in the dark. Cells were then analyzed using a FACSCalibur flow cytometer (BD Biosciences, United States), and data were processed using FlowJo software.

For apoptosis detection, the Annexin V‐FITC/Propidium Iodide (PI) Apoptosis Detection Kit (Bestbio, China) was used. After experimental treatments, cells were resuspended in binding buffer and incubated with Annexin V‐FITC and PI according to the manufacturer′s instructions. Samples were incubated at room temperature for 15 min in the dark. Cell populations were subsequently analyzed using a FACSCalibur flow cytometer, and the results were quantified with FlowJo software.

### 2.4. PDPN Knockdown by siRNA

Gene‐specific and negative control siRNAs targeting PDPN were purchased from Sigma‐Aldrich (United States). The target sequences for PDPN were as follows: siRNA: 5 ^′^‐CCACUCUGUGGACAAGAAATT‐3 ^′^; 5 ^′^‐UUUCUUGUCCACAGAGUGGTT‐3 ^′^. RAW264.7 cells were seeded in six‐well plates at a density of 1 × 10^6^ cells per well and allowed to adhere for 24 h. Transfection was performed using 100 pM of each siRNA mixed with 4 *μ*L of Lipo8000 transfection reagent (C0533, Beyotime, China) in serum‐free DMEM. After 6 h of incubation at 37°C in a 5% CO_2_ atmosphere, the transfection medium was replaced with fresh complete DMEM supplemented with 10% FBS. Cells were further cultured under standard conditions until subsequent experimental treatments were performed.

### 2.5. Cell Viability Assay

RAW264.7 cells were seeded into 96‐well plates at a density of 5 × 10^3^ cells per well and incubated for 24 h prior to treatment. Cell proliferation was assessed using the Cell Counting Kit‐8 (CCK‐8; M006, CTCC, China) following the manufacturer′s instructions. Briefly, 20 *μ*L of CCK‐8 solution was added to each well, mixed gently, and incubated for an additional 2 h at 37°C. Absorbance was measured at 450 nm using a microplate reader (MK3, Thermo, United States) to evaluate cell viability.

### 2.6. Western Blotting Analysis

**Figure 1:**
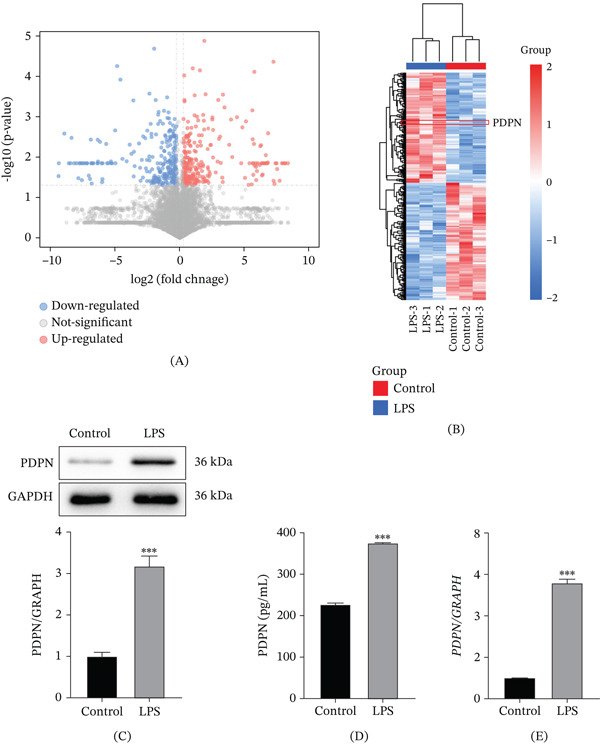
(A–E) LPS stimulation significantly upregulates PDPN expression in macrophages.

Cells were lysed in RIPA buffer (P0013B, Beyotime, China) supplemented with phenylmethylsulfonyl fluoride (PMSF, BP2655, RUIBIO, China) as a protease inhibitor. Total protein concentrations were quantified using a BCA Protein Assay Kit (BL521A, Biosharp, China). Equal amounts of protein samples were separated via SDS‐polyacrylamide gel electrophoresis and subsequently transferred onto polyvinylidene difluoride membranes (IPVH00010, Millipore, United States). Membranes were blocked with 5% bovine serum albumin for 1 h at room temperature to prevent nonspecific binding, followed by overnight incubation at 4°C with primary antibodies. The primary antibodies and their working dilutions were as follows: ERK (1:1000, 11257‐1‐AP, Proteintech, China), ERK phosphorylation (p‐ERK) (1:1000, 28733‐1‐AP, Proteintech, China), SERPINE1 (1:1000, 83980‐3‐RR, Proteintech, China), and GAPDH (1:2000, ab8245, Proteintech, China) as the internal control. After primary antibody incubation, membranes were washed and incubated for 1 h at room temperature with horseradish peroxidase (HRP)–conjugated secondary antibodies (anti‐mouse or anti‐rabbit IgG, 1:5000; ZSGB‐BIO, China). Protein bands were visualized using the ECL Prime Western Blotting Detection System (Dingguo Changsheng, China), and chemiluminescent signals were captured and quantified using the ChemiScope 5300 Pro imaging system (CLINX, China). Full‐length uncropped blots corresponding to Figures 1C, 3E, and 4F are provided in Supporting Information 1: Figure S1. Representative triplicate blots, densitometric quantification, and uncropped source images for the added revision‐stage assays are provided in Supporting Information 1: Figure S2 and the raw‐data package.

### 2.7. Enzyme‐Linked Immunosorbent Assay (ELISA) for PDPN, IL‐6, TNF‐*α*, and IL‐1*β*

The concentrations of PDPN and key proinflammatory cytokines, including IL‐6, TNF‐*α*, and IL‐1*β*, were quantified using ELISA kits (PDPN: YB‐70191H, Shanghai Yu Bo Biotech Co. Ltd., China; IL‐6: ml063159, TNF‐*α*: ml002095, IL‐1*β*: ml301814, mlbio, Shanghai, China). Briefly, cell culture supernatants were collected and transferred to 96‐well plates precoated with specific capture antibodies. The assay procedures were carried out in accordance with the manufacturer′s protocols. Absorbance was measured using a microplate reader, and cytokine concentrations were calculated based on standard curves.

### 2.8. Proteomic Analysis

#### 2.8.1. Preliminary Clinical Cohort Analysis

A preliminary clinical dataset including 57 patients with sepsis and 30 control individuals was analyzed during revision. Serum PDPN concentrations were measured by ELISA, and clinical variables, including SOFA score, APACHE II score, CRP, PCT, IL‐6, TNF‐*α*, and outcome, were collected from the deidentified dataset. Comparisons between groups and correlations between PDPN and clinical parameters were used to assess the translational relevance of PDPN in human sepsis.

The label‐free proteomic analysis workflow involves a structured process of data preprocessing, differential protein identification, and bioinformatics analysis. Initially, raw data undergoes normalization, peptide filtering, and missing value imputation, followed by Pearson′s correlation analysis to assess sample relationships. Differentially expressed proteins are identified based on statistical significance and fold change, serving as the basis for further analysis. Conventional analyses include PCA, volcano plots, clustering, and enrichment analyses (GO, KEGG, and COG), along with protein–protein interaction network construction. Advanced analyses such as multiomics integration and WGCNA are available upon request, with customizable visualization for publication purposes.

### 2.9. Statistical Analysis

All data are presented as mean ± standard deviation (SD). Two‐group comparisons were performed using unpaired two‐tailed Student′s *t*‐tests, and comparisons among three or more groups were performed using one‐way ANOVA followed by Tukey′s post hoc test. Correlations in the preliminary clinical cohort were analyzed using Spearman′s rank correlation. Statistical analyses were performed using GraphPad Prism 8 and Python 3. A two‐sided *p* value of less than 0.05 was considered statistically significant. No formal a priori sample‐size calculation was performed because the present work was exploratory in nature.

## 3. Results

### 3.1. LPS Stimulation Significantly Enhances PDPN Expression in Macrophages

To investigate the effects of LPS stimulation on PDPN expression in macrophages, we conducted comprehensive analyses at the proteomic, transcriptomic, and protein levels. Volcano plot analysis revealed significant differences in protein expression profiles between control and LPS‐treated macrophages, identifying numerous proteins that were either significantly upregulated or downregulated following LPS exposure (Figure 1A). Hierarchical clustering analysis further confirmed these observations by clearly distinguishing between control and LPS‐treated groups and identifying PDPN as markedly upregulated in macrophages treated with LPS (Figure 1B). Consistent with these proteomic findings, Western blot analysis demonstrated that PDPN protein levels were significantly increased in macrophages after LPS treatment compared to untreated controls (Figure 1C). Full‐length uncropped blots corresponding to Figures 1C, 3E, and 4F are provided in Supporting Information 1: Figure S1. Representative triplicate blots, densitometric quantification, and uncropped source images for the added revision‐stage assays are provided in Supporting Information 1: Figure S2 and the raw‐data package. Moreover, ELISA results validated these findings by showing significantly higher PDPN concentrations in the culture supernatants of LPS‐treated macrophages relative to controls (Figure 1D, *p* < 0.05). qPCR analysis further corroborated these protein‐level results, indicating a significant elevation of PDPN mRNA expression in response to LPS stimulation (Figure 1E, *p* < 0.05). Collectively, these results strongly indicate that LPS significantly upregulates PDPN expression in macrophages at both the transcriptional and translational levels.

### 3.2. PDPN Monoclonal Antibody SZ168 Alleviates Inflammation and Regulates Macrophage Viability, Polarization, and Apoptosis in LPS‐Treated Macrophages

**Figure 2:**
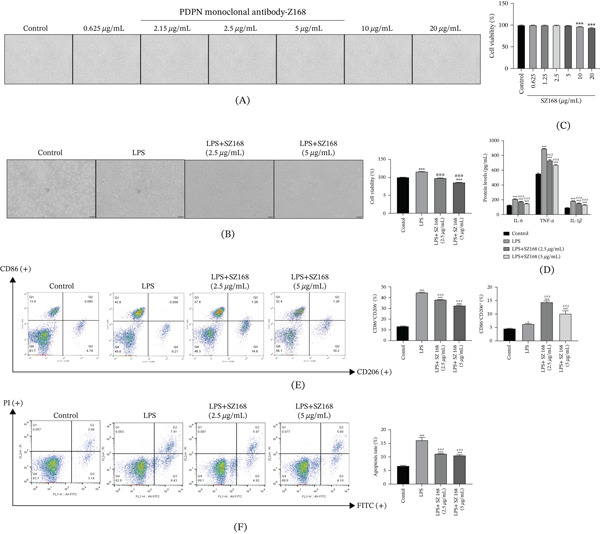
(A–F) PDPN monoclonal antibody SZ168 alleviates inflammatory responses, regulates macrophage polarization, and reduces viability and apoptosis in LPS‐stimulated macrophages.

To examine the biological effects of the PDPN monoclonal antibody SZ168 in LPS‐treated macrophages, we performed a series of cellular assays. First, we evaluated the cytotoxicity of SZ168 across a concentration range of 0.625–20 *μ*g/mL in RAW264.7 cells. No significant cytotoxic effects were observed at concentrations below 10 *μ*g/mL (Figure 2A), and concentrations of 2.5 and 5 *μ*g/mL were therefore selected for subsequent experiments. Under inflammatory conditions induced by LPS, macrophage viability increased markedly, whereas SZ168 at both 2.5 and 5 *μ*g/mL modestly reduced viability compared with the LPS group (Figure 2B, all *p* < 0.05). Western blotting further confirmed that LPS markedly increased PDPN protein abundance, while SZ168 attenuated this increase (Figure 2C). In parallel, ELISA analysis demonstrated that LPS stimulation dramatically increased the secretion of IL‐6, TNF‐*α*, and IL‐1*β*, and that SZ168 significantly reduced these cytokines, indicating attenuation of the inflammatory response (Figure 2D, all *p* < 0.05).

Flow cytometric analysis was subsequently performed to examine macrophage polarization. LPS treatment resulted in a significant increase in proinflammatory M1 macrophages (CD86^+^CD206^−^ cells), whereas SZ168 markedly reduced the proportion of CD86‐positive cells (Figure 2E, all *p* < 0.05). Conversely, SZ168 treatment significantly increased the population of anti‐inflammatory M2 macrophages (CD86^−^CD206^+^), indicating promotion of M2 polarization (Figure 2E, all *p* < 0.05). Apoptosis assays further revealed that LPS significantly elevated macrophage apoptosis rates, while SZ168 treatment notably decreased apoptosis levels (Figure 2F, all *p* < 0.05).

Collectively, these results demonstrate that the PDPN monoclonal antibody SZ168 effectively mitigates LPS‐induced inflammation, promotes anti‐inflammatory M2 polarization, and reduces apoptosis in macrophages exposed to LPS.

### 3.3. Proteomic Analysis Identifies Molecular Targets Regulated by PDPN Monoclonal Antibody SZ168 in LPS‐Treated Macrophages

**Figure 3:**
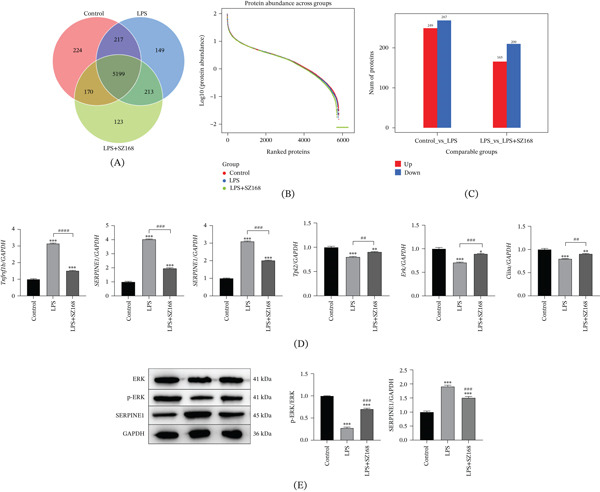
(A–E) Proteomic and molecular analyses identify molecular targets regulated by PDPN monoclonal antibody SZ168 in LPS‐treated macrophages.

To identify the molecular mechanisms underlying the effects of the PDPN monoclonal antibody SZ168 on macrophages treated with LPS, we performed label‐free proteomic analysis. A Venn diagram illustrated the overlap and differences in protein expression among control, LPS, and LPS + SZ168 groups (Figure 3A). Protein abundance profiles across these groups were presented, indicating overall similar distribution but also highlighting specific differentially expressed proteins induced by LPS and modulated by SZ168 treatment (Figure 3B). Differential protein expression analysis revealed that LPS treatment induced marked changes in protein expression, with a total of 249 proteins significantly upregulated and 267 downregulated compared with controls. Treatment with SZ168 reversed these effects, with 165 proteins upregulated and 209 downregulated relative to the LPS group (Figure 3C).

To further validate these findings, qPCR was performed to measure mRNA expression levels of selected candidate genes (Tnfrsf1b, SERPINE1, Tpl2, Erk, and Ciita). As shown in Figure 3D, LPS stimulation significantly increased the mRNA expression of Tnfrsf1b, SERPINE1, and Tpl2 and decreased the expression of Erk and Ciita compared to the control group (all *p* < 0.05). SZ168 treatment notably reversed these transcriptional changes (all *p* < 0.05). Additionally, Western blot analysis confirmed the changes observed at the protein level. LPS treatment significantly reduced p‐ERK and elevated SERPINE1 expression, whereas SZ168 treatment markedly reversed these alterations (Figure 3E, all *p* < 0.05).

Taken together, these results demonstrate that SZ168 modulates specific molecular pathways and gene expression profiles altered by LPS in macrophages, highlighting ERK signaling and SERPINE1 as key molecular targets regulated by SZ168 treatment.

#### 3.3.1. Additional MEK, p90RSK, and c‐Fos Validation Further Supports ERK Pathway Involvement

To extend mechanistic validation beyond ERK and SERPINE1, we measured additional upstream and downstream mediators of the ERK axis. As shown in Supporting Information 1: Figure S2, LPS decreased MEK, p90RSK, c‐Fos, and p‐ERK/ERK, whereas SZ168 partially restored all four signals. PDPN knockdown further enhanced the effects of SZ168, resulting in the highest p‐ERK/ERK, MEK/GAPDH, p90RSK/GAPDH, and c‐Fos/GAPDH levels together with the lowest SERPINE1/GAPDH level in the LPS + siRNA‐PDPN + SZ168 group. These additional data strengthen the conclusion that SZ168 alleviates inflammatory dysregulation through coordinated modulation of the ERK signaling cascade.

### 3.4. PDPN Knockdown Confirms the Protective Effects of PDPN Monoclonal Antibody SZ168 via ERK Signaling in LPS‐Treated Macrophages

To further elucidate the functional role of PDPN in mediating the protective effects of SZ168 in macrophages stimulated with LPS, PDPN expression was knocked down using siRNA. qPCR analysis confirmed successful PDPN knockdown by three different PDPN‐specific siRNAs (siRNA‐176, siRNA‐234, and siRNA‐477), among which siRNA‐234 demonstrated the highest knockdown efficiency and was thus selected for subsequent experiments (Figure 4A, *p* < 0.05). We then assessed macrophage viability under various experimental conditions. LPS stimulation significantly increased macrophage viability compared to the control group (*p* < 0.05), whereas SZ168 treatment notably decreased cell viability (*p* < 0.05). Interestingly, PDPN knockdown further enhanced the adverse effects of SZ168 on macrophage viability, suggesting that PDPN downregulation potentiates SZ168‐mediated effects in cell survival (Figure 4B, all *p* < 0.05).

Figure 4(A–D) PDPN knockdown enhances the anti‐inflammatory, antiapoptotic, and macrophage polarization‐modulating effects of PDPN monoclonal antibody SZ168 via ERK signaling in LPS‐treated macrophages.

**Figure:**
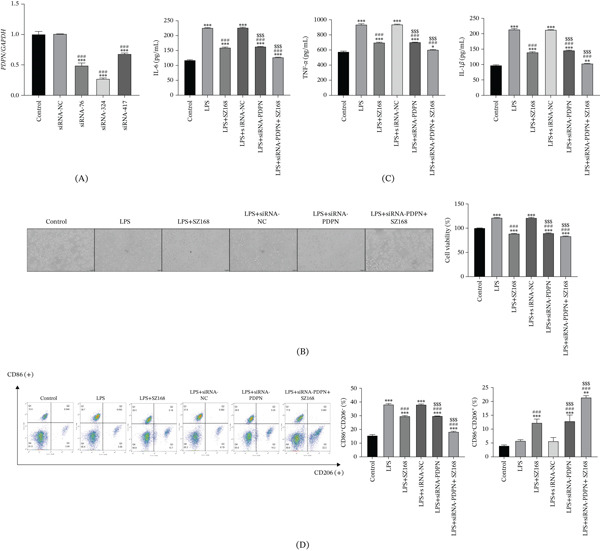

**Figure:**
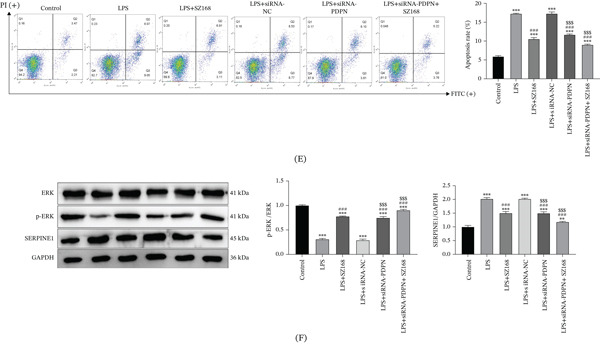

ELISA analysis demonstrated that LPS stimulation markedly increased the secretion of proinflammatory cytokines (IL‐6, TNF‐*α*, and IL‐1*β*). SZ168 significantly suppressed these inflammatory cytokines, an effect further augmented by PDPN knockdown, underscoring the critical involvement of PDPN in regulating inflammation (Figure 4C, all *p* < 0.05). Flow cytometric analysis revealed that PDPN knockdown enhanced SZ168‐mediated modulation of macrophage polarization under inflammatory conditions. Specifically, knockdown of PDPN further reduced the population of proinflammatory M1 macrophages (CD86^+^CD206^−^) while significantly increasing anti‐inflammatory M2 macrophages (CD86^−^CD206^+^), indicating that PDPN is crucial for SZ168‐regulated macrophage polarization (Figure 4D, all *p* < 0.05). Additionally, apoptosis assays revealed that SZ168 markedly reduced LPS‐induced apoptosis, and PDPN knockdown significantly strengthened this antiapoptotic effect, highlighting the importance of PDPN in SZ168‐mediated apoptosis regulation (Figure 4E, all *p* < 0.05).

Finally, Western blot analysis confirmed that PDPN knockdown significantly enhanced SZ168‐induced restoration of p‐ERK and suppression of SERPINE1 expression in LPS‐treated macrophages. These results identify the ERK signaling pathway as a key mechanism through which PDPN mediates SZ168′s beneficial effects (Figure 4F, all *p* < 0.05).

Collectively, these findings strongly support the conclusion that PDPN is central to the anti‐inflammatory, polarization–modulatory, and antiapoptotic effects of SZ168 in LPS‐treated macrophages, acting primarily through regulation of the ERK signaling pathway.

#### 3.4.1. Supplementary In Vivo Validation Shows That SZ168 Ameliorates Sepsis‐Related Organ Injury and Macrophage Polarization

**Figure 5:**
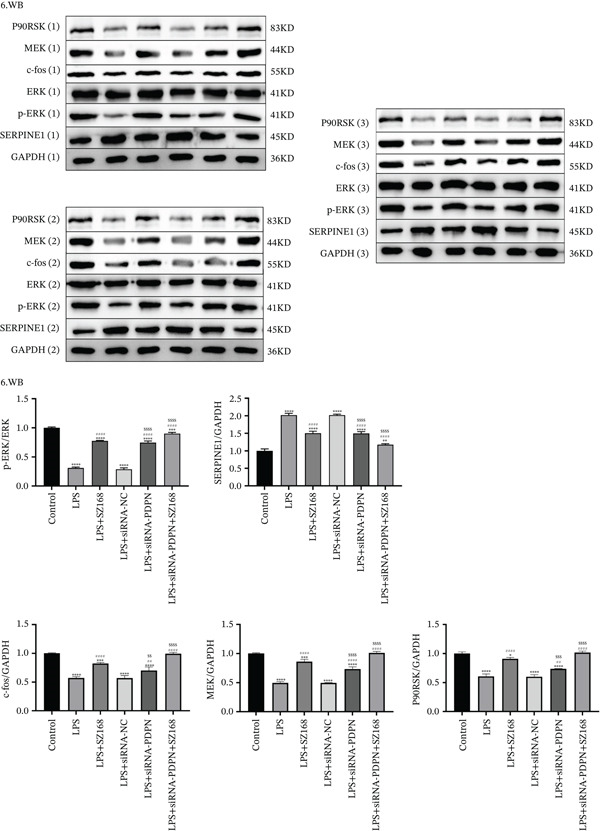
SZ168 activated the ERK signaling pathway in cells that were inhibited by LPS.

**Figure 7:**
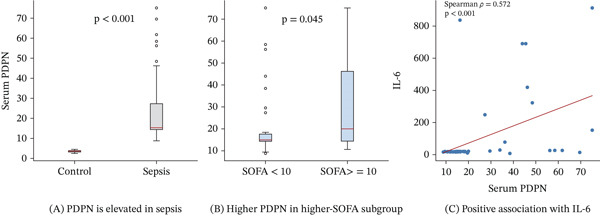
(A–C) Elevated serum PDPN is associated with disease severity and inflammation in patients with sepsis.

To address the concern that the original manuscript was largely cell‐based, we analyzed supplementary in vivo mouse data obtained during revision (Supporting Information 1: Figure S3). Compared with the model group, SZ168 reduced BUN and creatinine; decreased IL‐1*β*, IL‐6, and TNF‐*α* in both organ supernatants and serum; and ameliorated lung histopathological injury. Immunofluorescence staining further showed reduced F4/80 + CD86^+^ signals and increased F4/80 + CD206^+^ signals after SZ168 treatment, consistent with suppression of proinflammatory macrophage activation and promotion of an anti‐inflammatory phenotype in vivo (Figures 5, 6, and 7).

Figure 6SZ168 mitigates organ injury, suppresses inflammation, and promotes M2 macrophage polarization in a mouse sepsis model.

**Figure:**
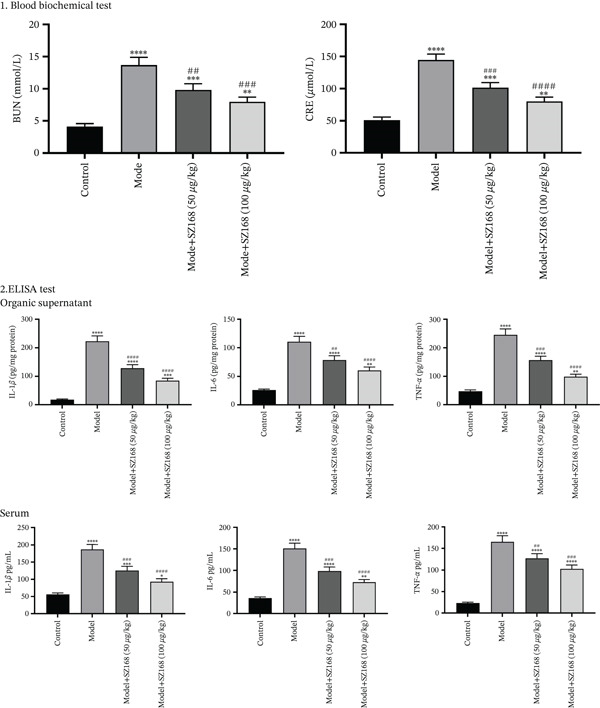

**Figure:**
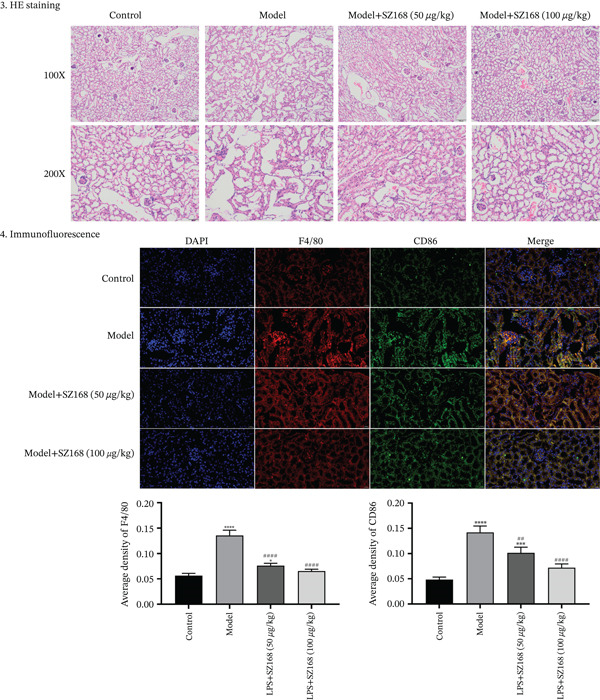

**Figure:**
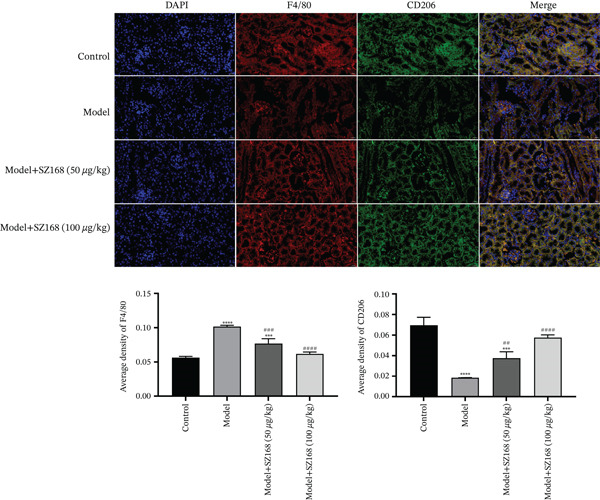

#### 3.4.2. Preliminary Human Data Support Elevated PDPN Expression in Sepsis

In the preliminary clinical cohort, serum PDPN levels were significantly higher in patients with sepsis than in controls (23.81 ± 17.64 vs. 3.53 ± 0.58, *p* < 0.001; Supporting Information 1: Figure S4). Among patients with sepsis, those with SOFA scores ≥ 10 had higher PDPN levels than those with SOFA scores < 10 (32.39 ± 21.74 vs. 20.16 ± 14.40, *p* = 0.045). Spearman′s analysis further showed a positive correlation between PDPN and IL‐6 (rho = 0.572, *p* < 0.001). These clinical data provide preliminary translational support for PDPN dysregulation in human sepsis.

## 4. Discussion

Sepsis remains a significant global health challenge, characterized by a dysregulated immune response that leads to systemic inflammation, multiple organ dysfunction, and high mortality rates [1–3]. Despite advances in therapeutic strategies targeting infection control and inflammation, effective treatments capable of modulating immune dysregulation during sepsis are still urgently needed. Macrophages, essential components of innate immunity, have increasingly been recognized for their pivotal roles in the development and progression of sepsis. The ability of macrophages to polarize into proinflammatory M1 or anti‐inflammatory M2 phenotypes significantly influences inflammatory responses, immune homeostasis, and outcomes during septic conditions [8–11]. Therefore, identifying novel molecular targets involved in macrophage polarization and function may provide valuable insights into the pathogenesis and potential therapeutic strategies for sepsis.

In this study, we focused on PDPN, a mucin‐type transmembrane glycoprotein known primarily for its role in platelet aggregation and immune regulation via CLEC‐2 interactions [12]. Although PDPN is generally minimally expressed in resting macrophages, its expression is notably induced during inflammatory states, including sepsis [13, 14]. Previous studies suggest that PDPN–CLEC‐2 interactions facilitate macrophage recruitment to infection sites, potentially enhancing bacterial clearance [15]. Yet, the specific roles of PDPN in macrophage function, polarization, and signaling pathways during septic conditions have remained incompletely understood.

Our comprehensive experimental design first established that PDPN expression is significantly elevated in macrophages upon LPS stimulation, confirmed by proteomic, transcriptomic, and protein‐level analyses. These data support prior findings indicating that PDPN is a key molecule involved in macrophage responses to inflammatory stimuli [17]. Next, we explored the biological significance of PDPN upregulation by utilizing a specific PDPN monoclonal antibody, SZ168, previously characterized for its ability to block PDPN–CLEC‐2 interactions [18]. We demonstrated that SZ168 significantly reduced inflammatory cytokine secretion, modulated macrophage polarization toward an anti‐inflammatory (M2) phenotype, and inhibited apoptosis under inflammatory conditions.

To elucidate the molecular mechanisms underlying the protective effects of SZ168, we performed proteomic analyses followed by validation at both the mRNA and protein levels. These analyses identified significant alterations in key inflammatory and regulatory pathways, notably ERK signaling and the expression of SERPINE1 and related inflammatory mediators (Tnfrsf1b, SERPINE1, Tpl2, Erk, and Ciita) [19–23]. During revision, we further extended pathway validation to MEK, p90RSK, and c‐Fos, which changed in the same direction as p‐ERK and were most strongly restored in the combined si‐PDPN and SZ168 group. Thus, coordinated modulation of the ERK cascade appears to be a major mechanism through which PDPN influences macrophage inflammatory responses during sepsis.

To further validate PDPN′s involvement, we employed siRNA‐mediated PDPN knockdown. Intriguingly, PDPN knockdown markedly enhanced the anti‐inflammatory and antiapoptotic effects of SZ168, further promoting macrophage polarization toward the anti‐inflammatory M2 phenotype. The supplementary in vivo findings added during revision, together with the preliminary human cohort analysis, support the biological relevance of this axis beyond the original cell‐based system. These data strengthen the view that PDPN is critically involved in macrophage functional regulation during inflammation and that targeting PDPN provides protection against inflammatory damage, partly through ERK signaling modulation.

Nevertheless, several limitations should still be considered. First, although supplementary in vivo mouse data and a preliminary human cohort analysis were added during revision, the mechanistic core of this study remains largely cell‐based. Second, direct SZ168‐PDPN binding assays, PDPN‐knockout models, time‐course signaling analyses, and ERK rescue experiments were not included in the current revision and will be important for future mechanistic confirmation. Third, a cecal ligation and puncture model would further strengthen the translational relevance of the in vivo findings. Finally, the human dataset was modest in size and should be validated in larger prospective cohorts.

In conclusion, this study identifies PDPN as a key regulator of macrophage inflammation, polarization, and apoptosis in septic conditions. Our results demonstrate that the PDPN monoclonal antibody SZ168 mitigates inflammatory responses and apoptosis while promoting anti‐inflammatory macrophage polarization through ERK signaling modulation. The additional pathway, in vivo, and preliminary human data incorporated during revision further support PDPN as a promising therapeutic target for alleviating macrophage‐mediated inflammatory damage in sepsis.

## Funding

No funding was received for this manuscript.

## Conflicts of Interest

The authors declare no conflicts of interest.

## Data Availability Statement

The deidentified clinical dataset and experimental source data generated and/or analyzed during the current study are not publicly available due to institutional data‐protection regulations but are available from the corresponding authors upon reasonable request.

## Supporting Information

Additional supporting information can be found online in the Supporting Information section.

## Supporting information


**Supporting Information 1**   

**Supporting Information 2**   
