# Supplementary material for: Podoplanin Antibody SZ168 Alleviates Sepsis Inflammation and Macrophage Dysregulation via ERK Signaling
Source: Hum Mutat. 2026 Jun 8;2026:3791421. doi: 10.1155/humu/3791421 (PMC13247308; doi:10.1155/humu/3791421)

**Revision Supplementary Additions**

Supplementary Figure S1 remains the original uncropped-blot PDF (Supplementary File.pdf). The present document collects the additional materials organized during revision.

**Supplementary Figure S2. Additional ERK-axis validation after revision.**

Representative triplicate Western blots and densitometric quantification for p-ERK/ERK, SERPINE1/GAPDH, c-Fos/GAPDH, MEK/GAPDH, and p90RSK/GAPDH across the Control, LPS, LPS + SZ168, LPS + siRNA-NC, LPS + siRNA-PDPN, and LPS + siRNA-PDPN + SZ168 groups.


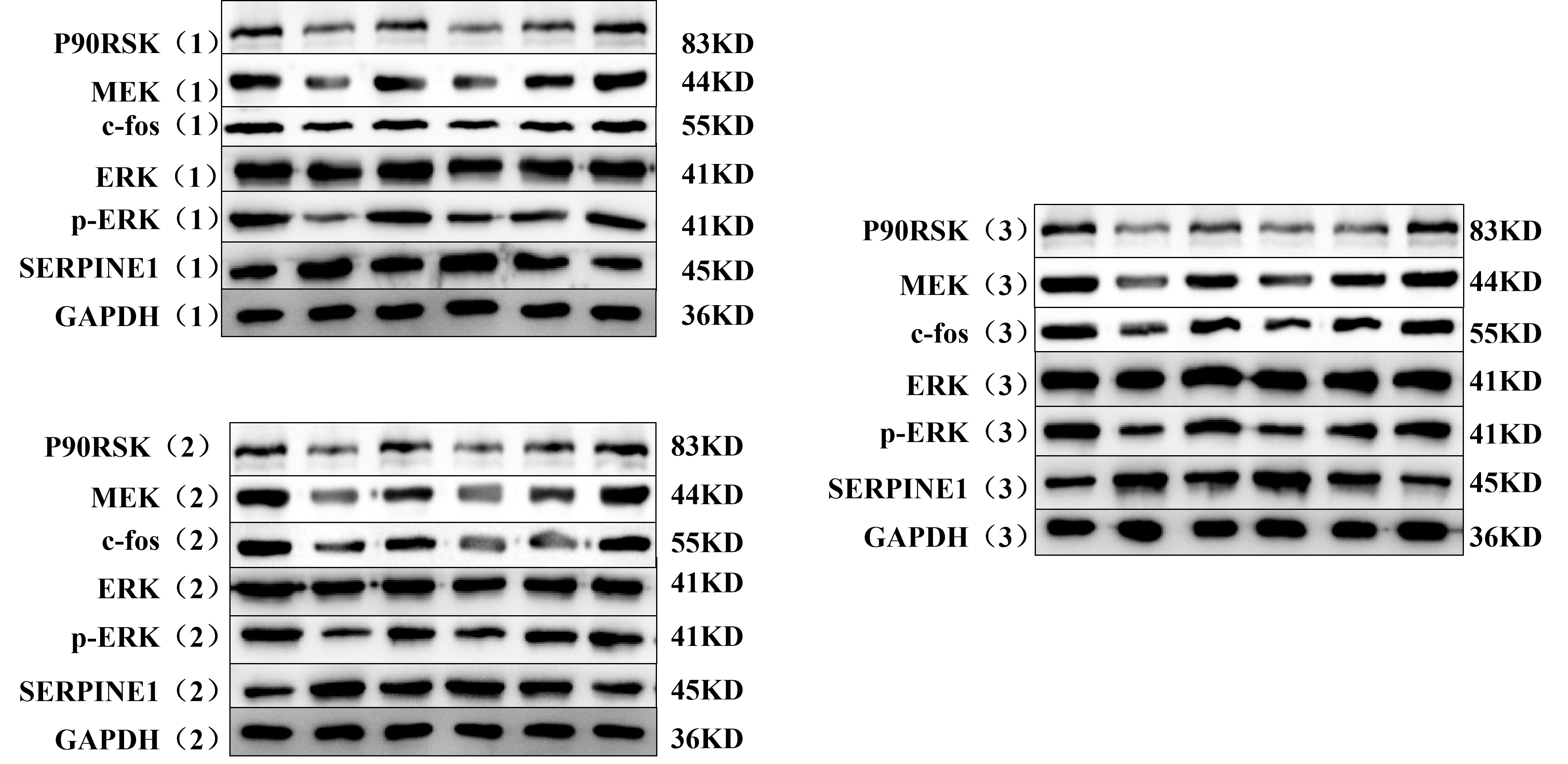


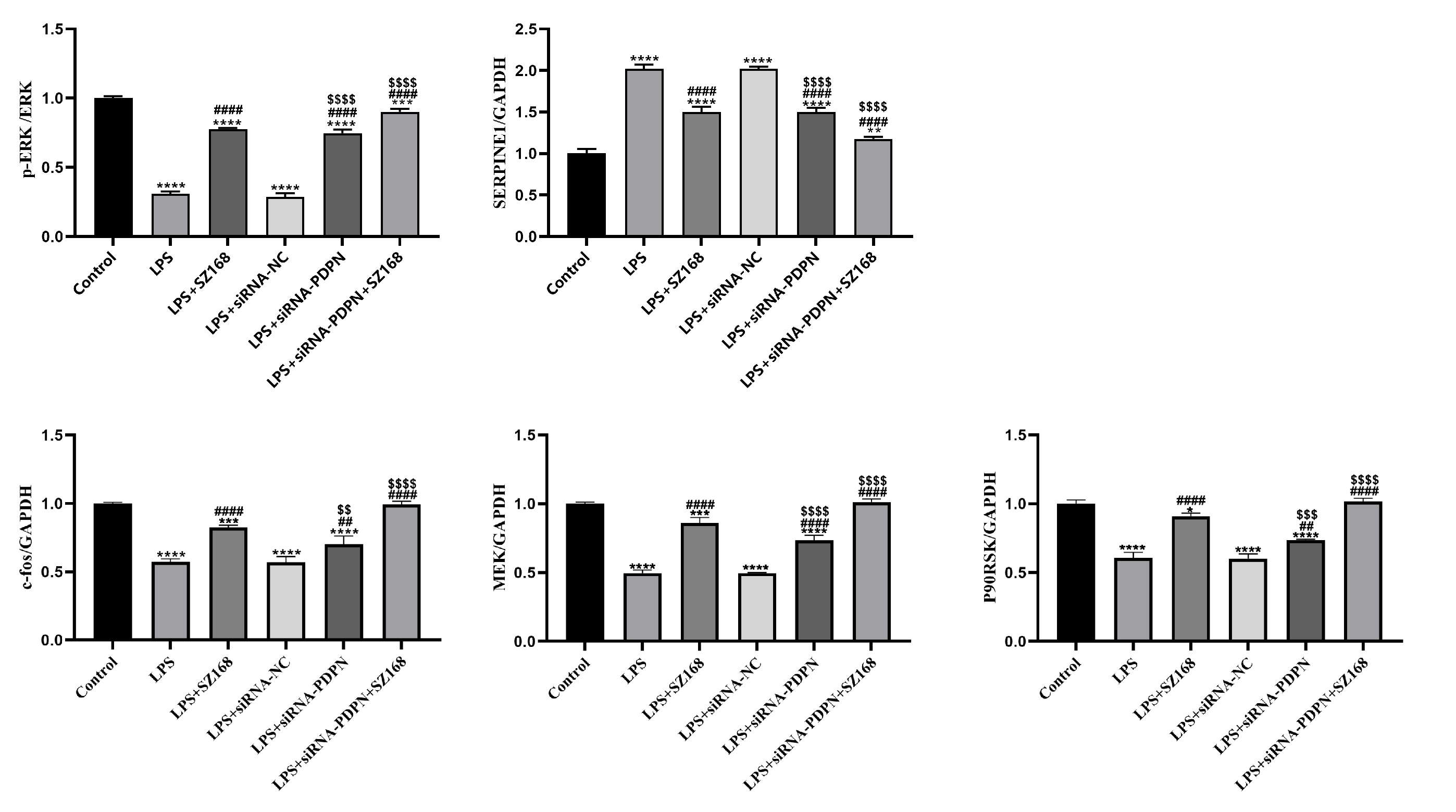


**Supplementary Figure S3. Supplementary in vivo validation of SZ168 in a mouse sepsis model.**

Additional mouse data available in the folder show improved renal injury markers, lower inflammatory cytokines, reduced lung injury by H&E staining, and a shift from F4/80+CD86+ toward F4/80+CD206+ signals after SZ168 treatment.


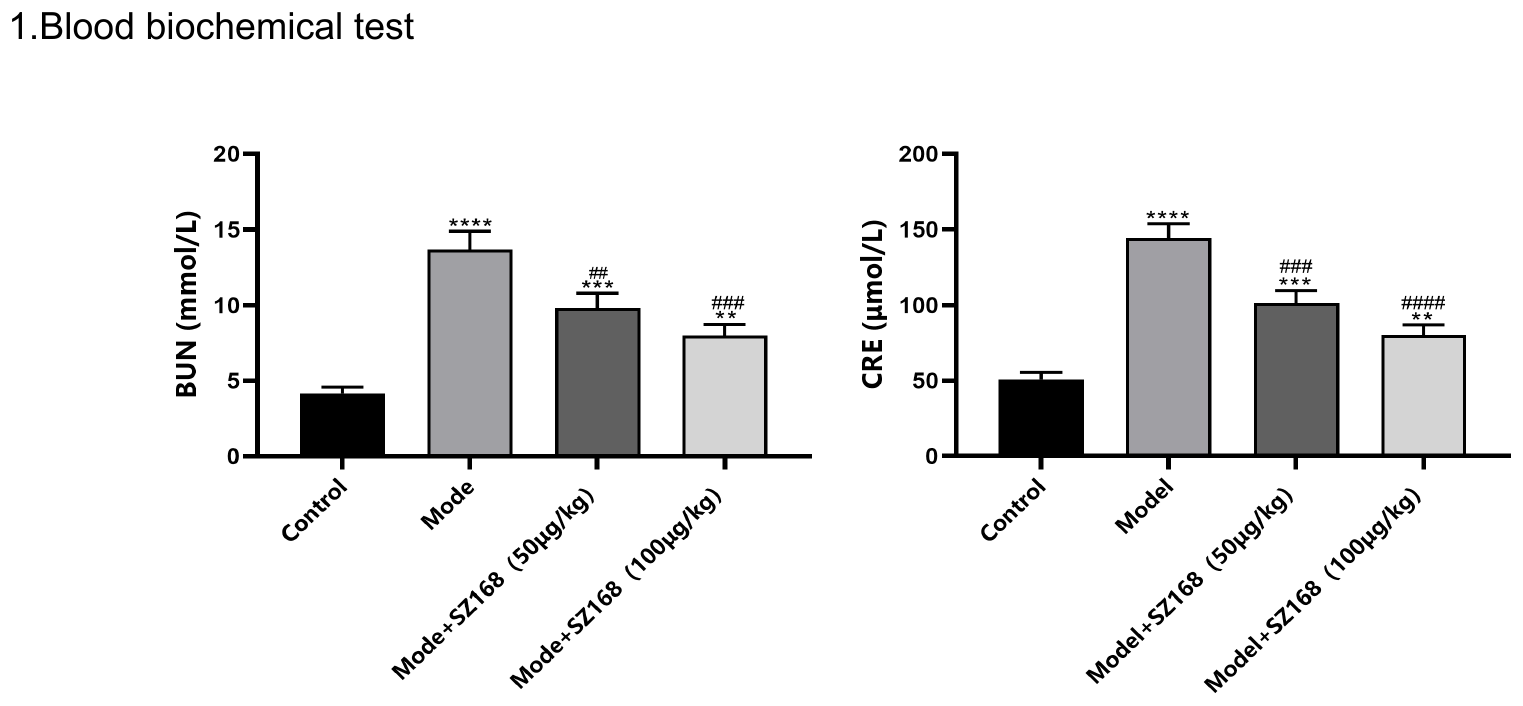


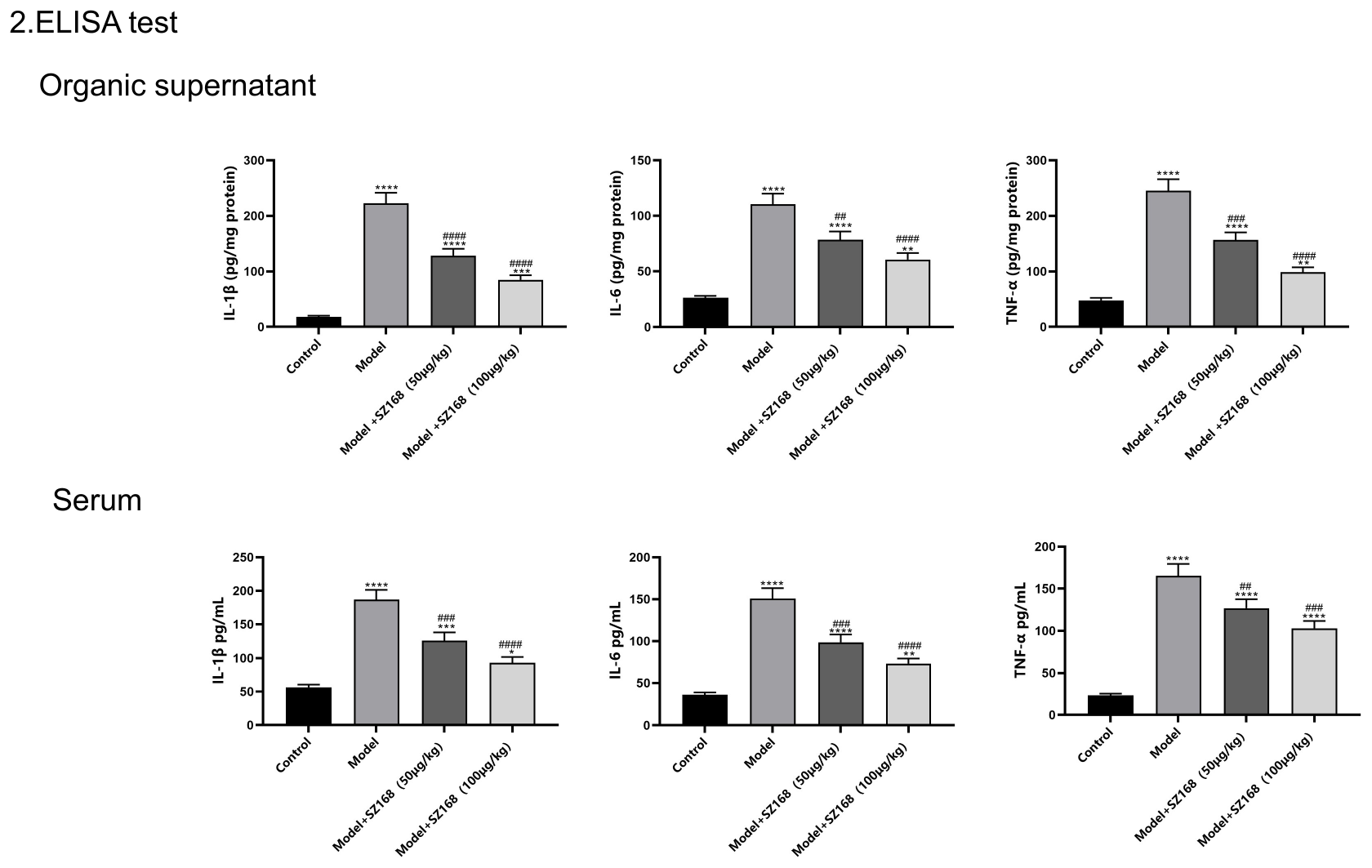


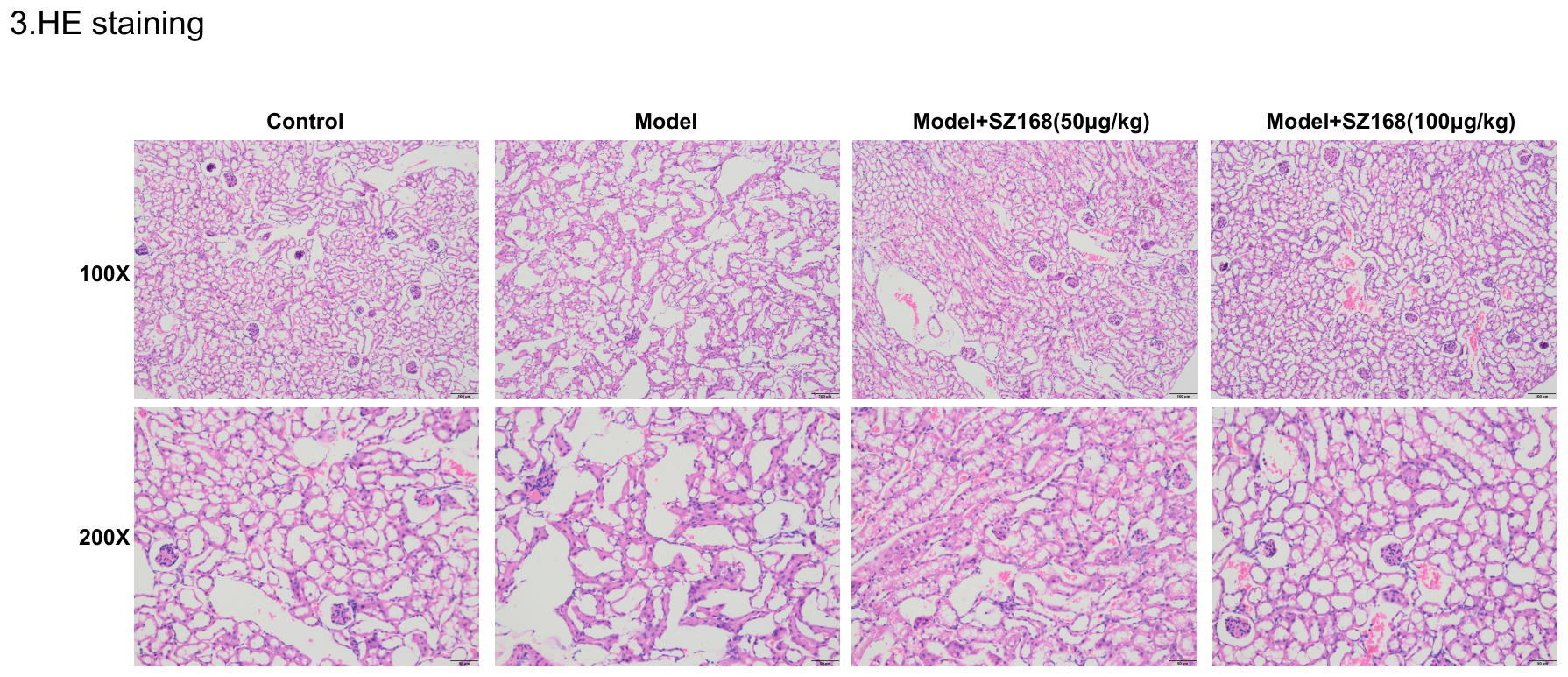


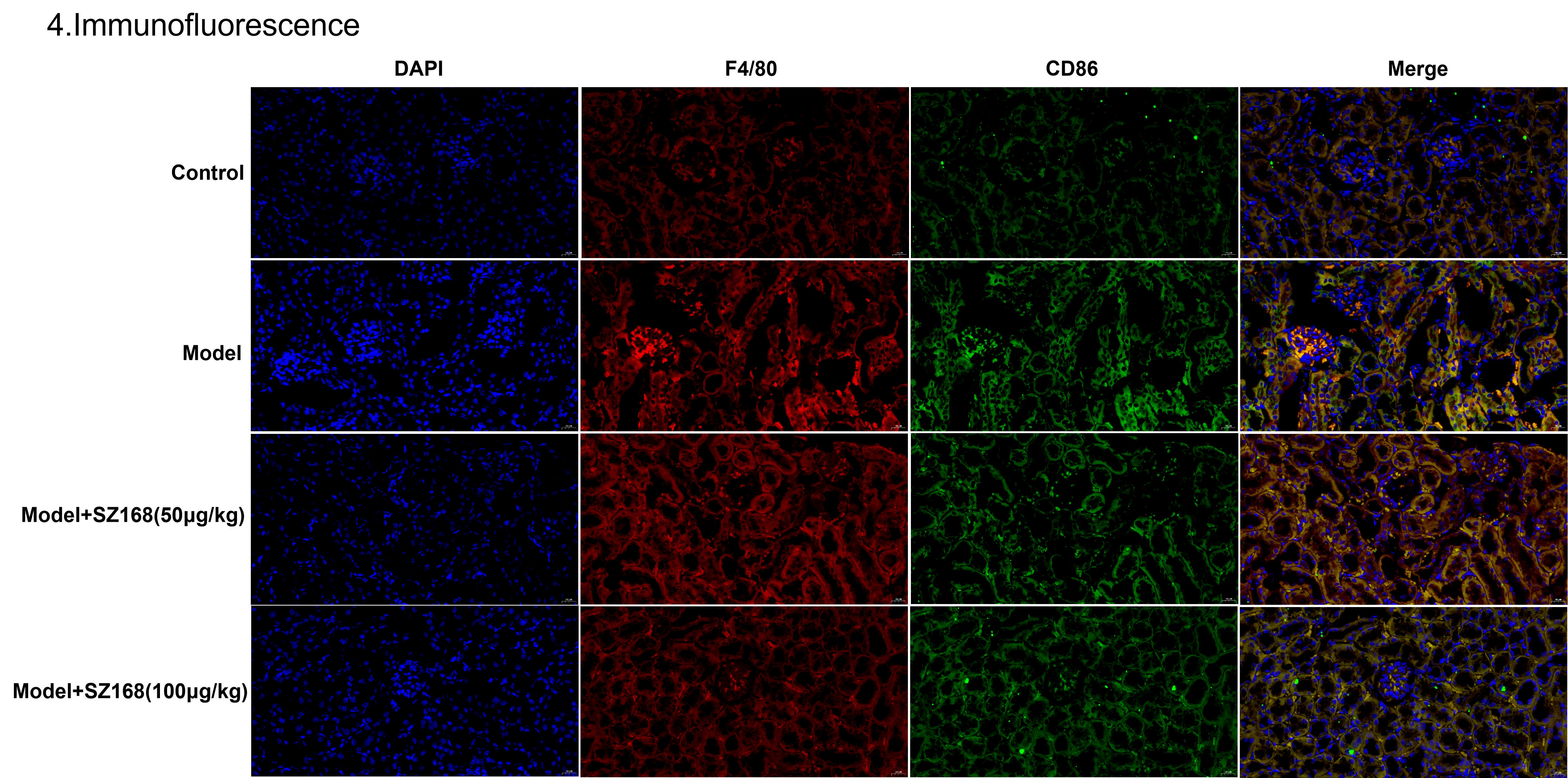


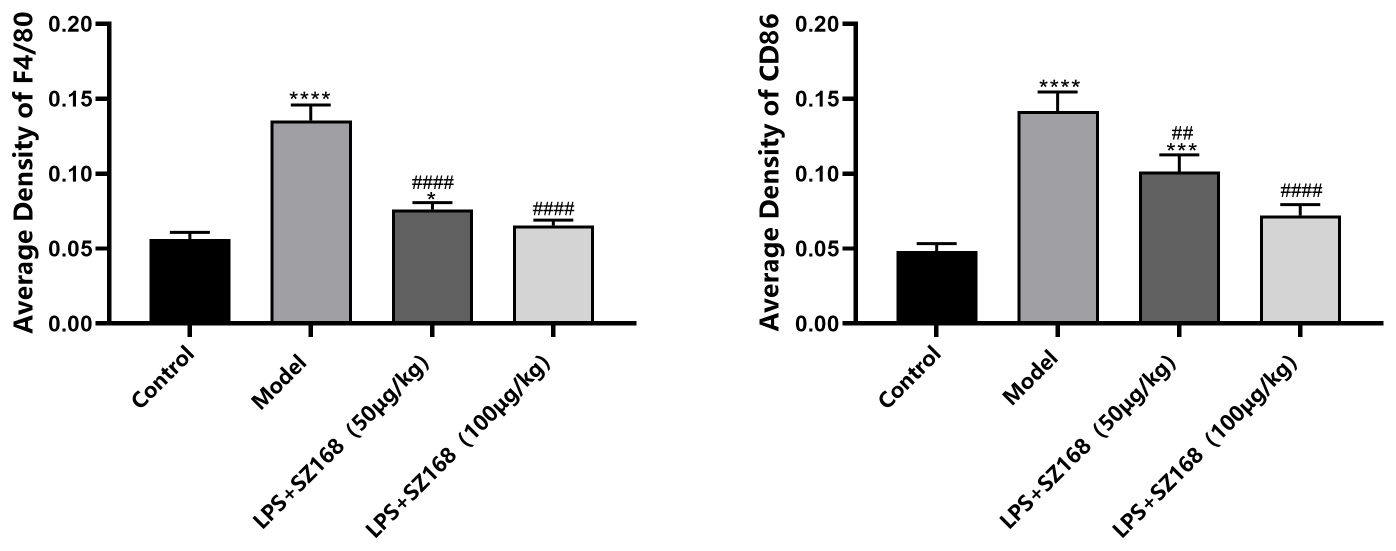


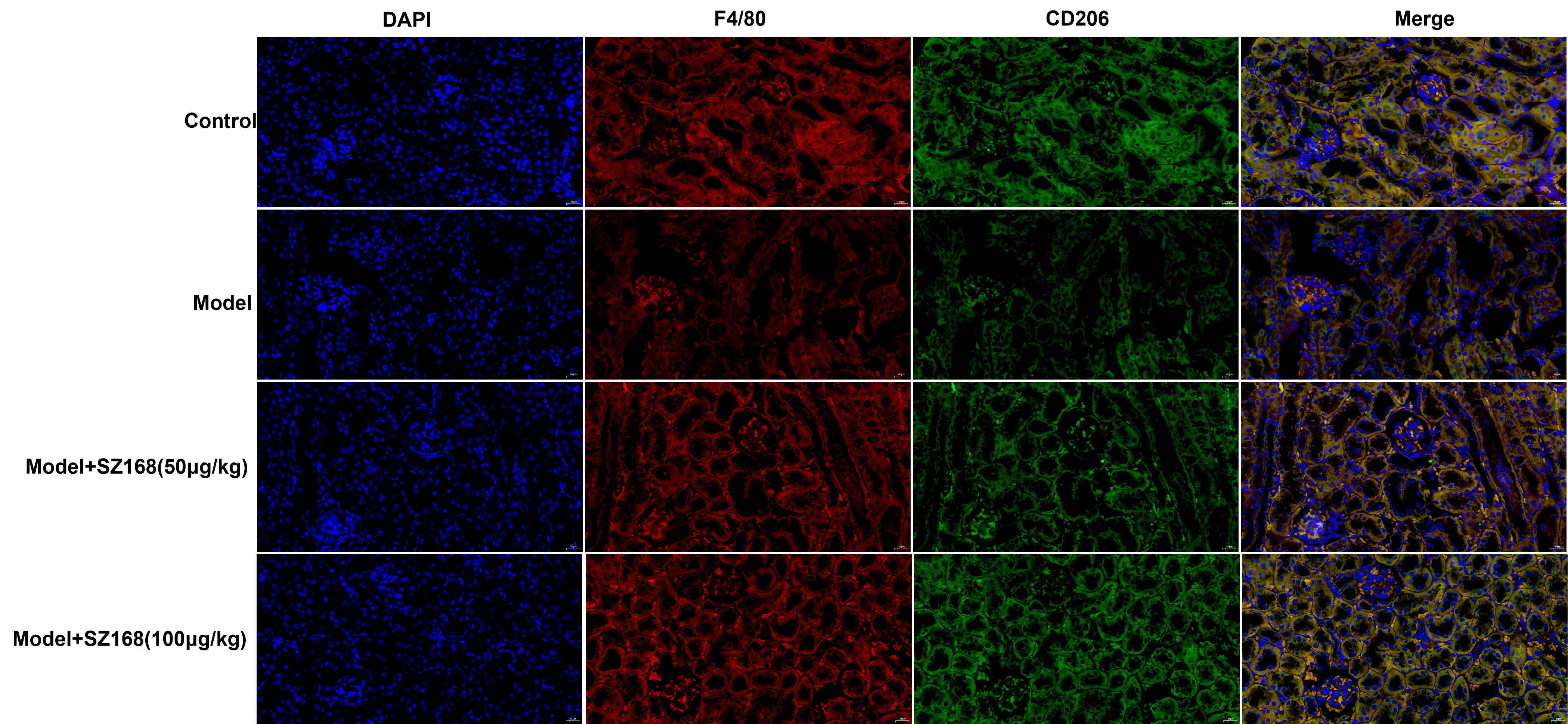


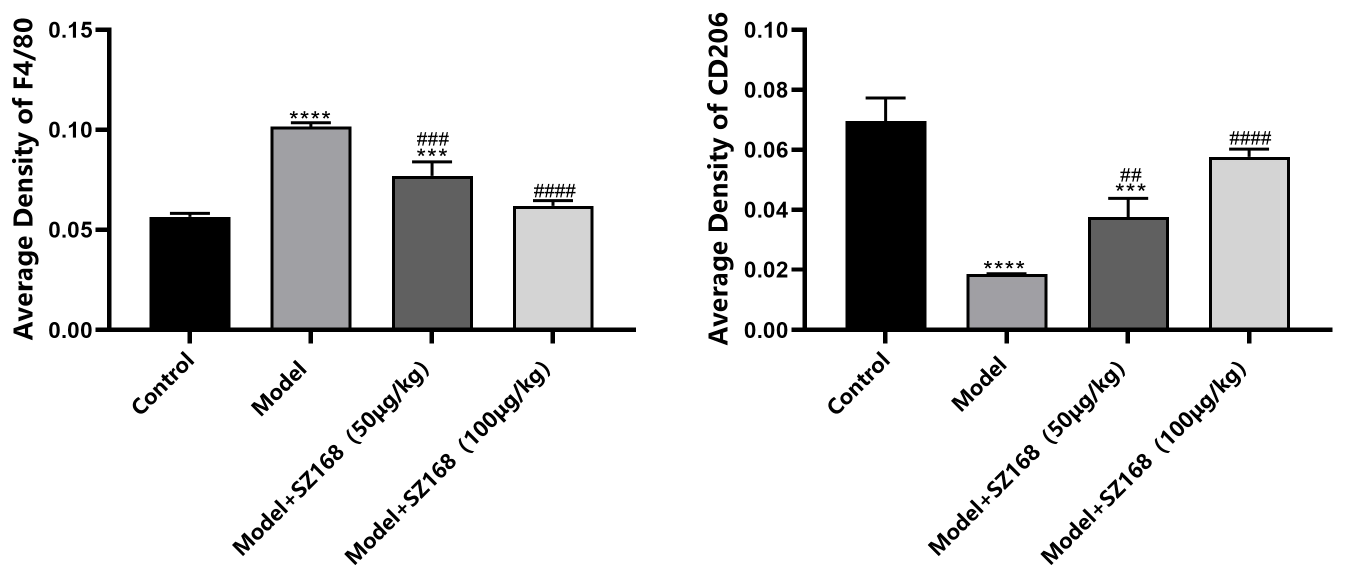


**Supplementary Figure S4. Preliminary clinical relevance of PDPN in human sepsis.**

Serum PDPN was higher in sepsis than in controls, increased in the higher-SOFA subgroup, and showed a positive association with IL-6. Cohort sizes: sepsis n = 57, control n = 30.


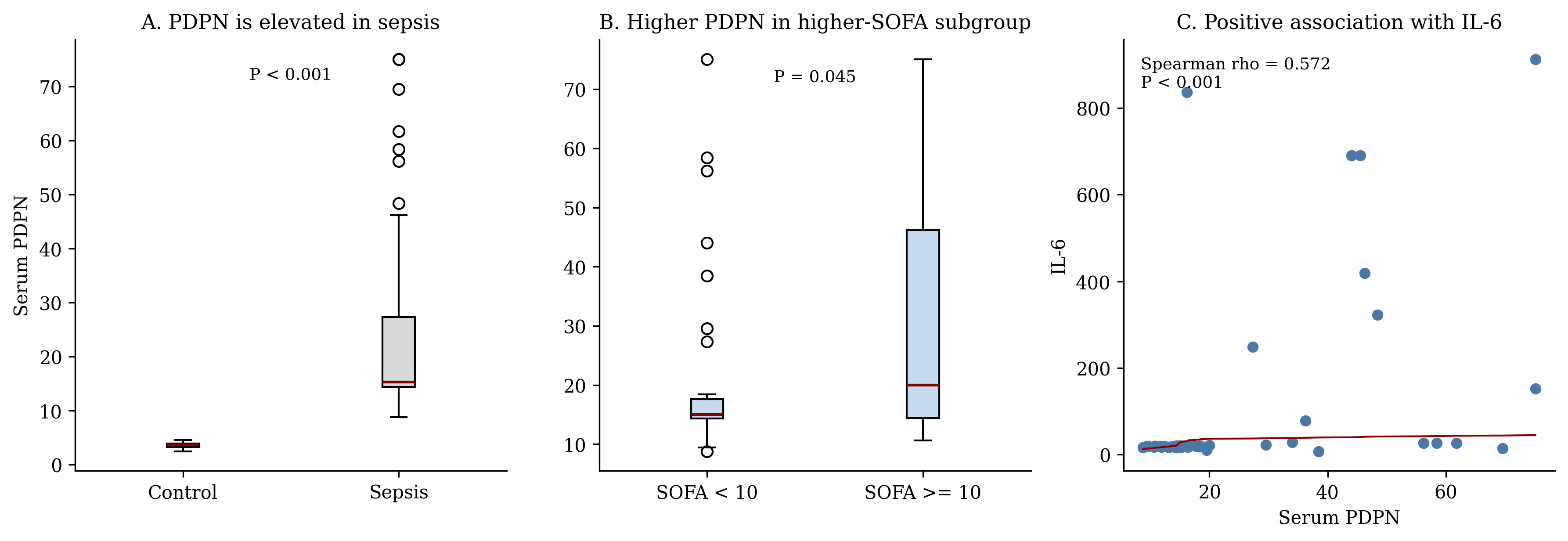

Supplement: Supplementary file 1 — Supporting Information 1 [file HUMU-2026-3791421-s001.docx]
