# Supplementary material for: Podoplanin Antibody SZ168 Alleviates Sepsis Inflammation and Macrophage Dysregulation via ERK Signaling
Source: Hum Mutat. 2026 Jun 8;2026:3791421. doi: 10.1155/humu/3791421 (PMC13247308; doi:10.1155/humu/3791421)
Supplement: Supplementary file 2 — Supporting Information 2 [file HUMU-2026-3791421-s002.pdf]

Figure 1 C

Uncropped Blots:

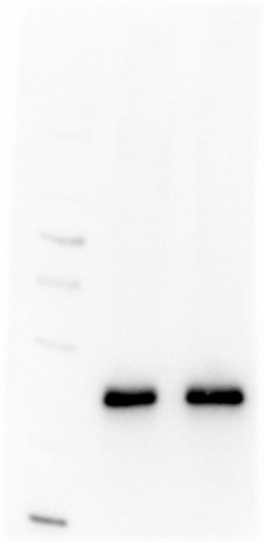

GAPDH (1)

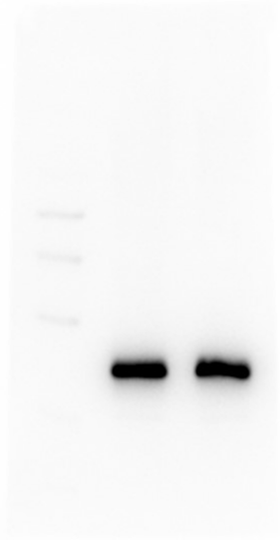

GAPDH (2)

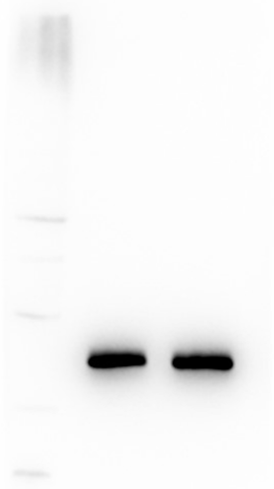

GAPDH (3)

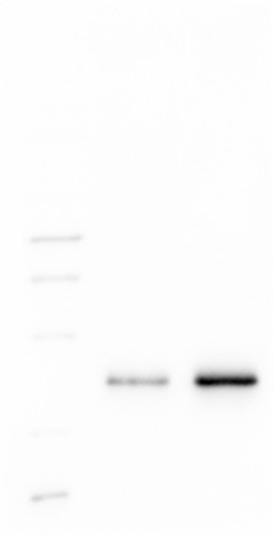

PDPN (1)

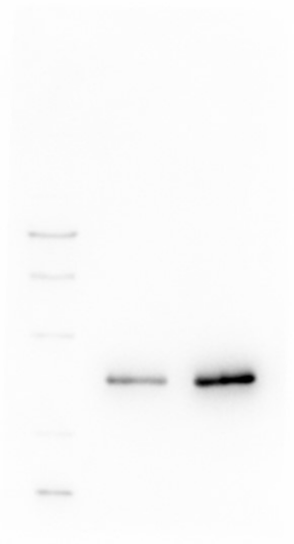

PDPN (2)

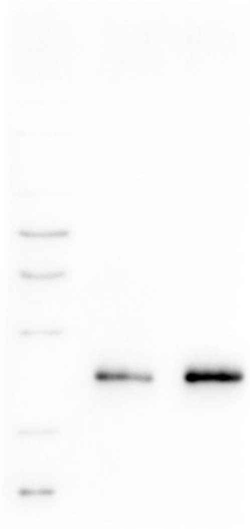

PDPN (3)

Cropped blots:

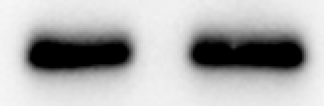

GAPDH (1)

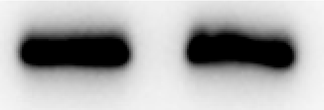

GAPDH (2)

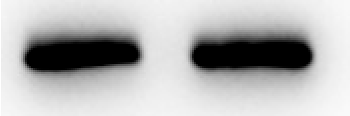

GAPDH (3)

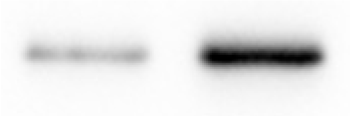

PDPN (1)

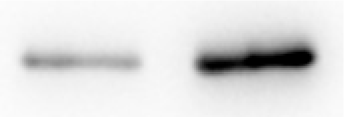

PDPN (2)

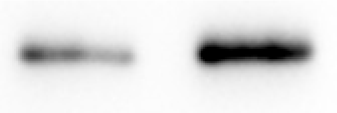

PDPN (3)

Figure 3 E

Uncropped Blots:

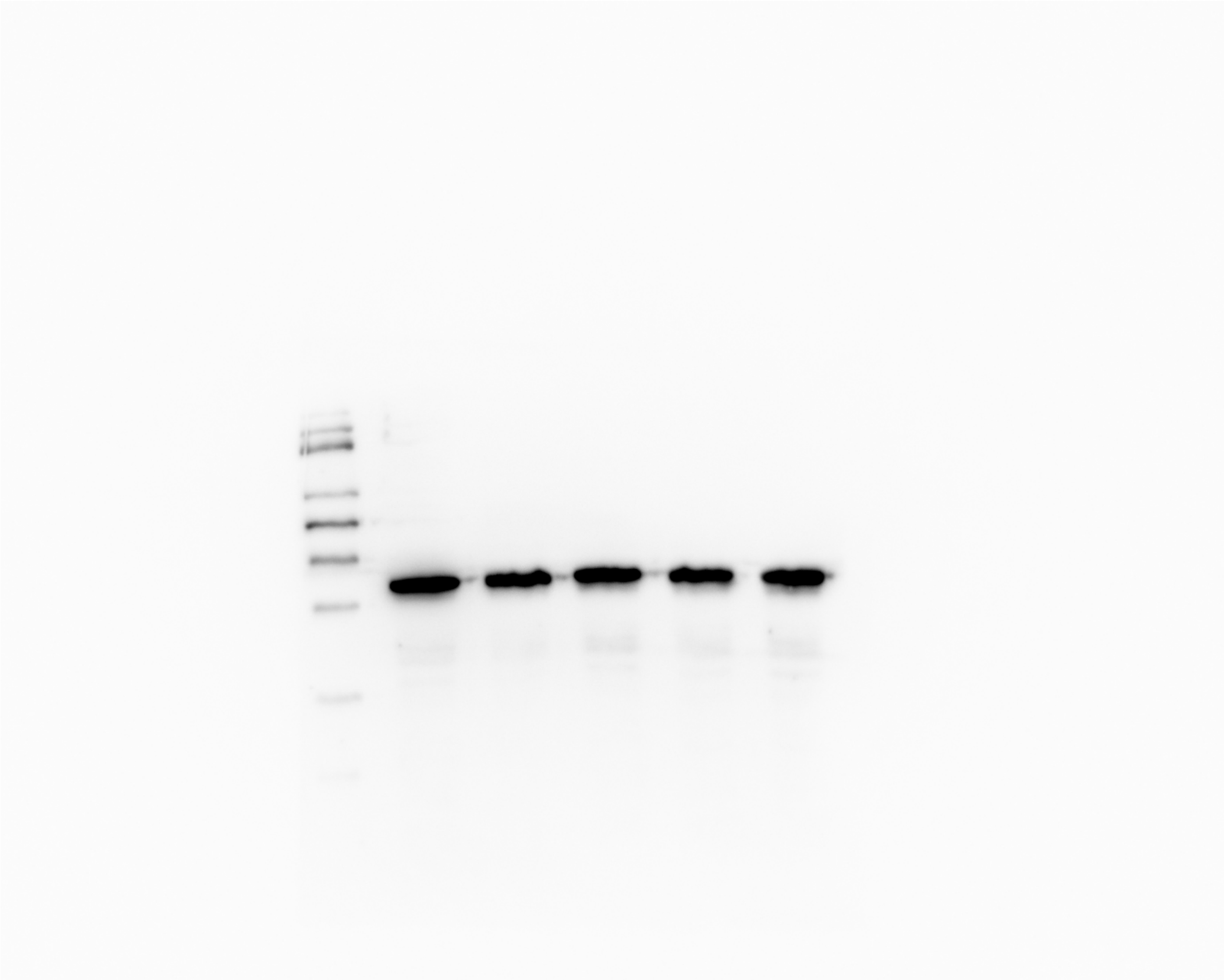

ERK-1

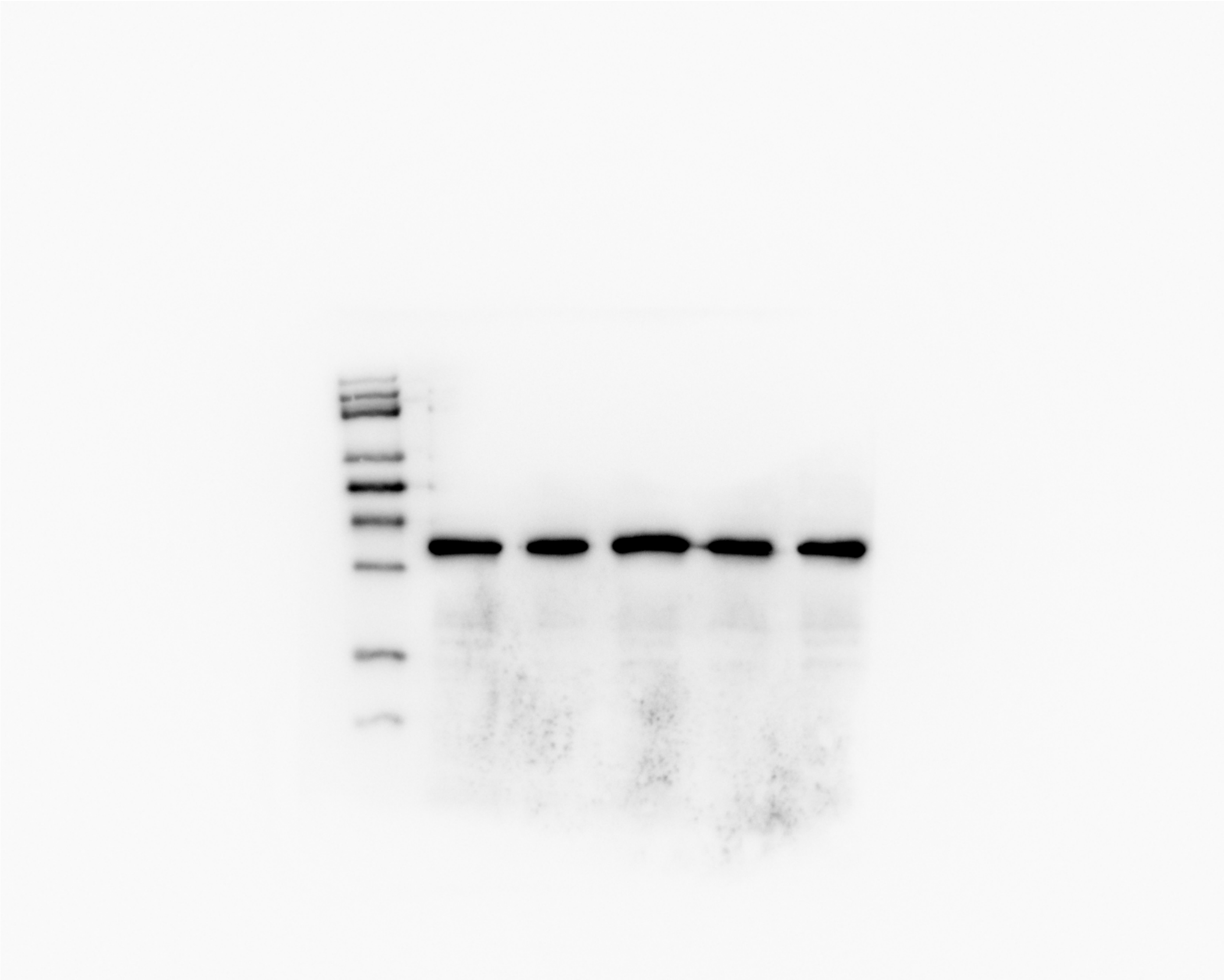

ERK-2

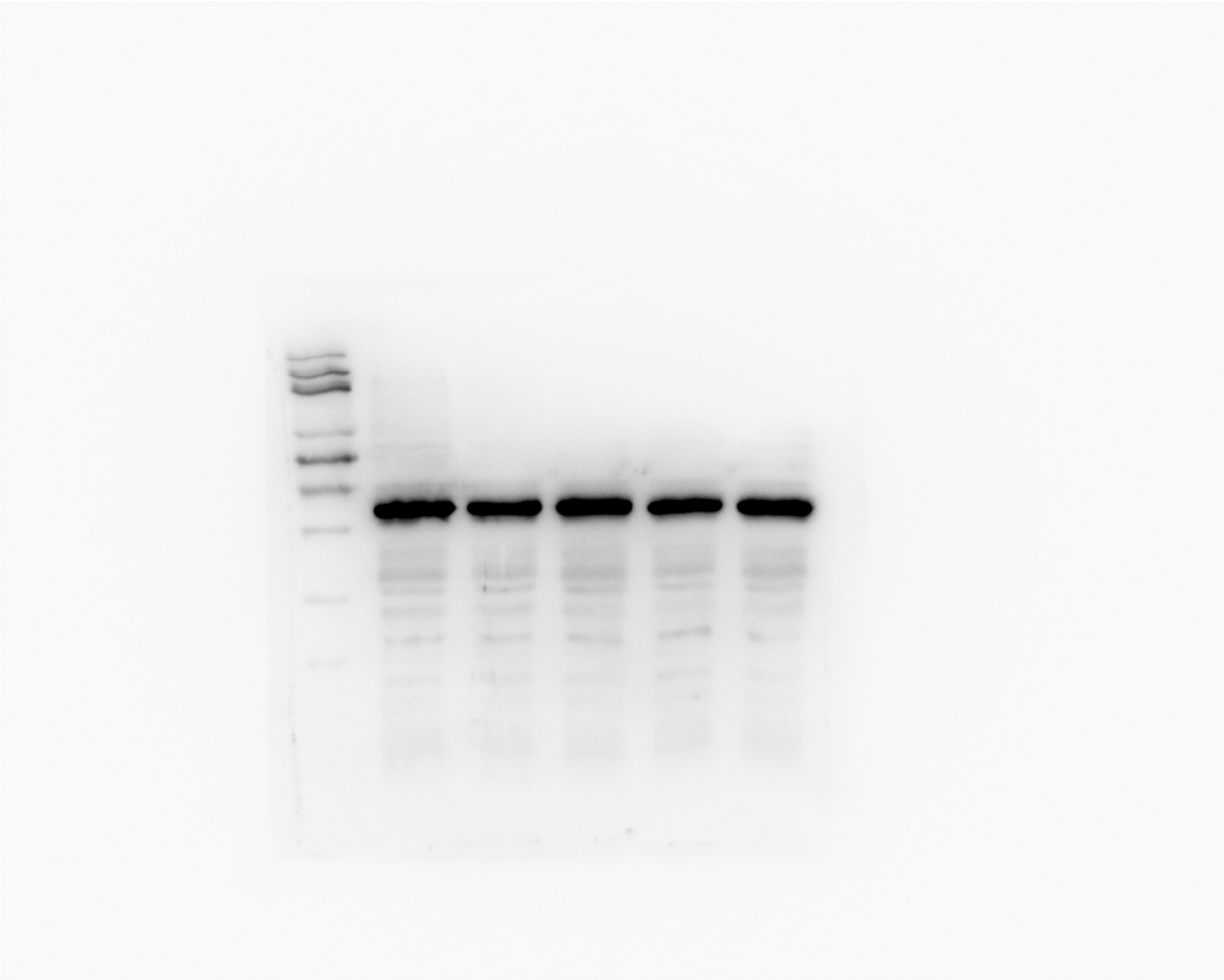

ERK-3

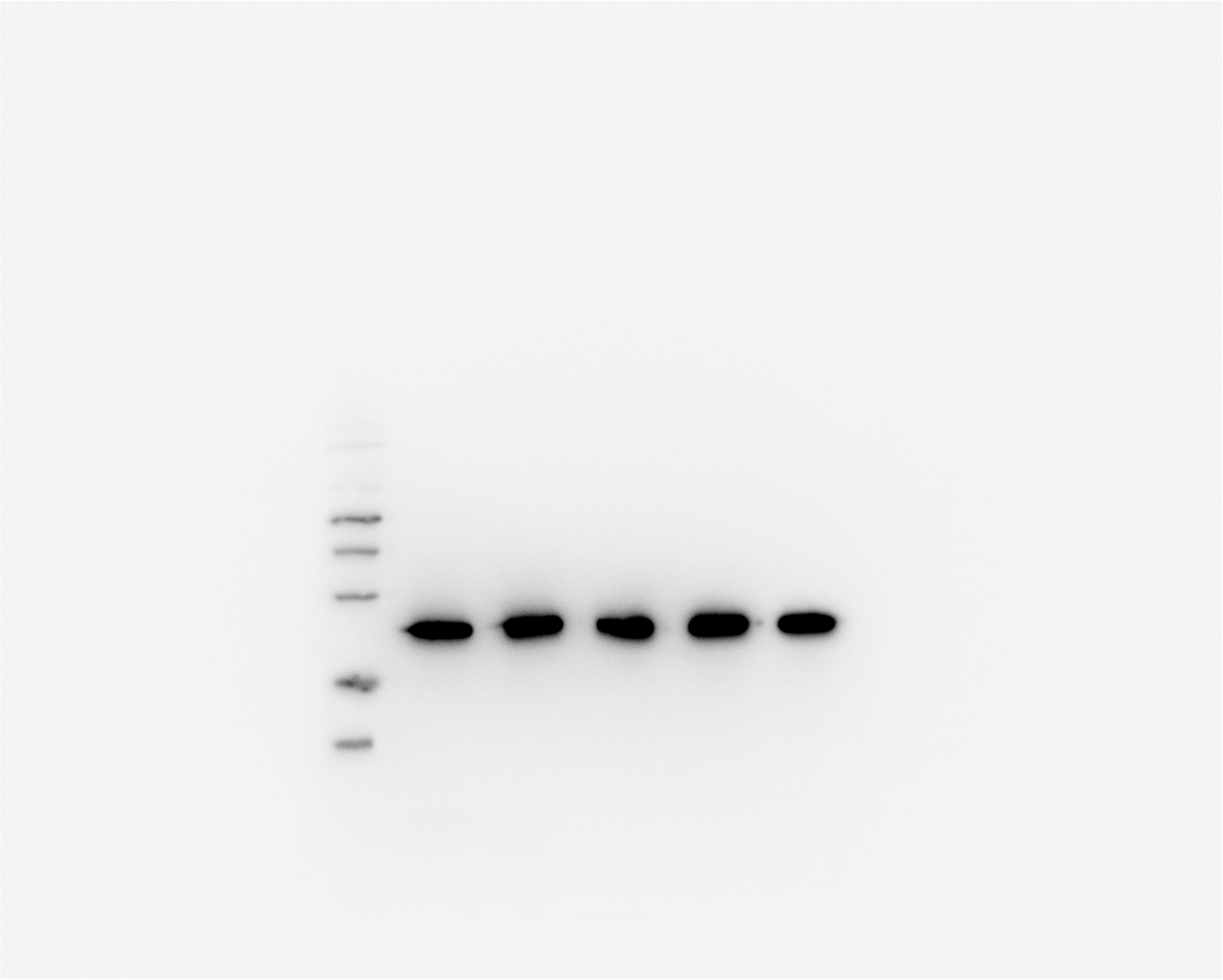

GAPDH-1

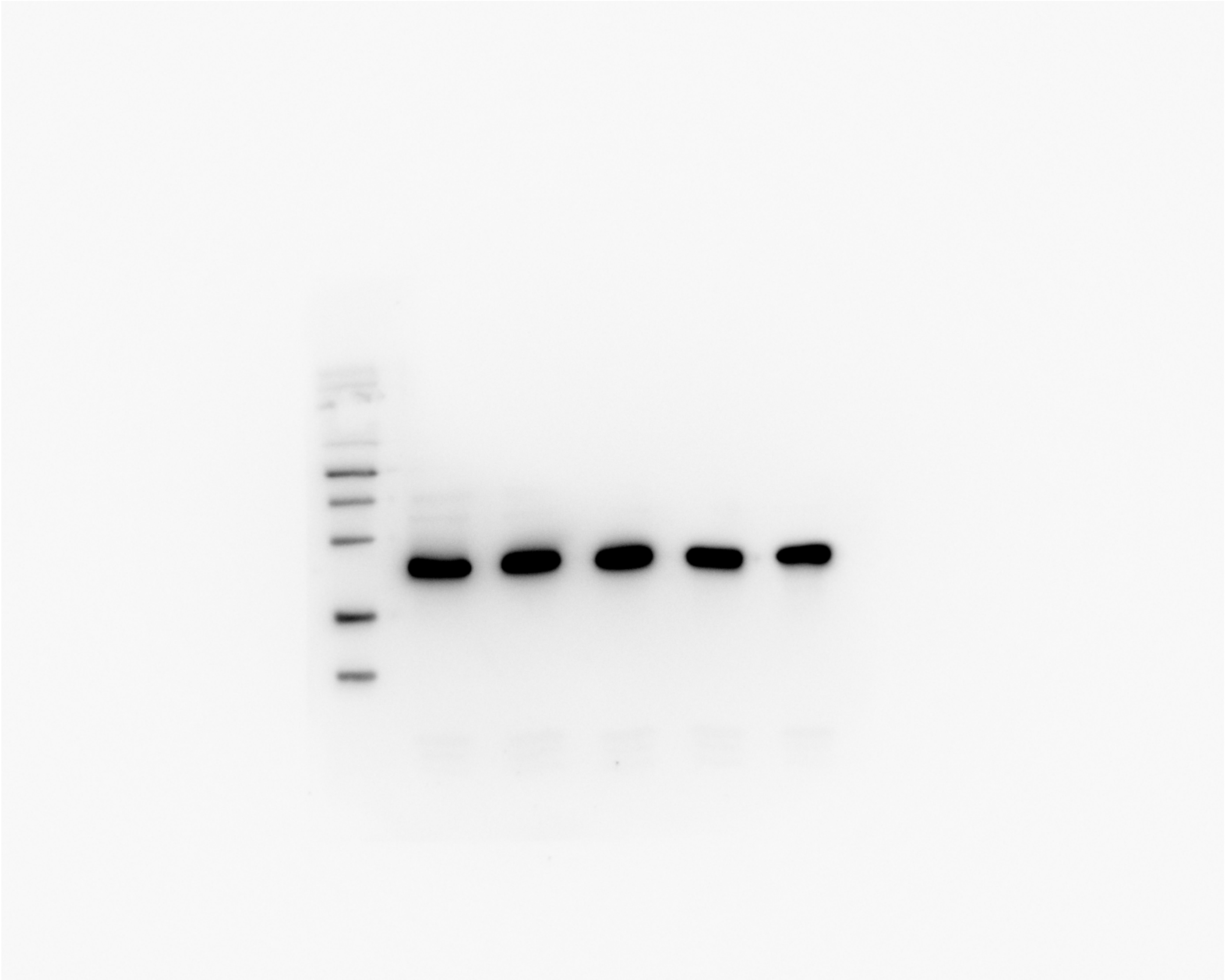

GAPDH-2

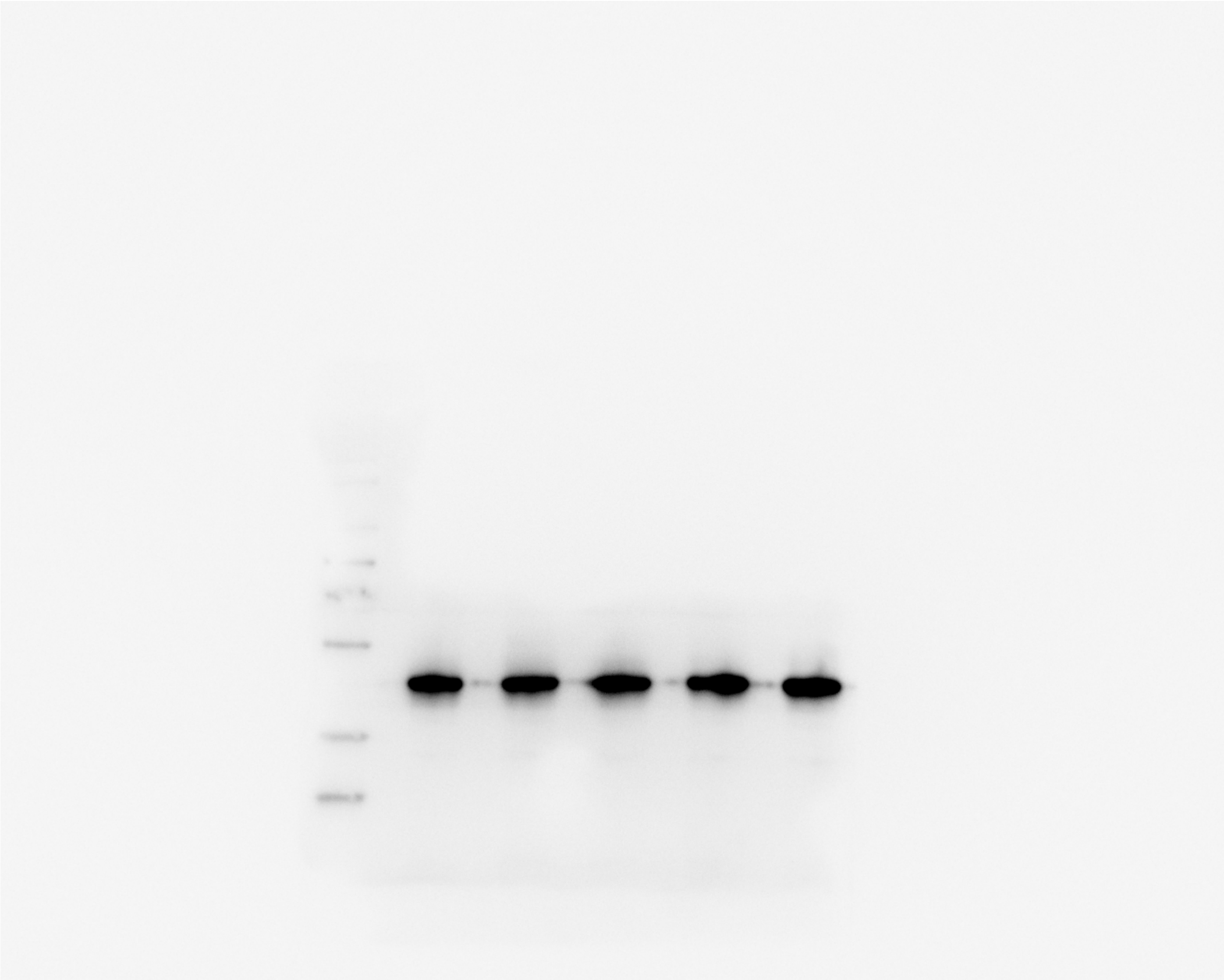

GAPDH-3

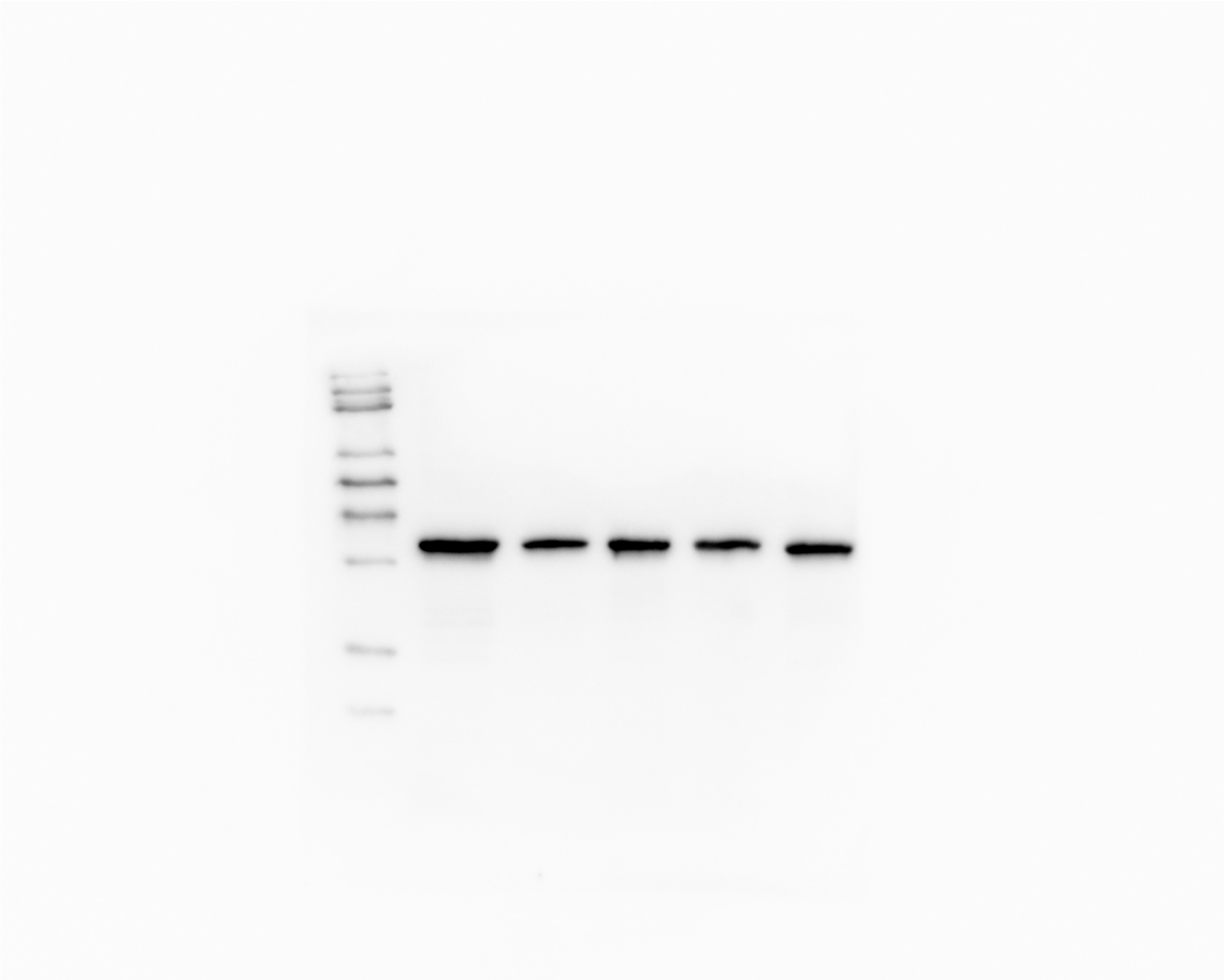

P-ERK-1

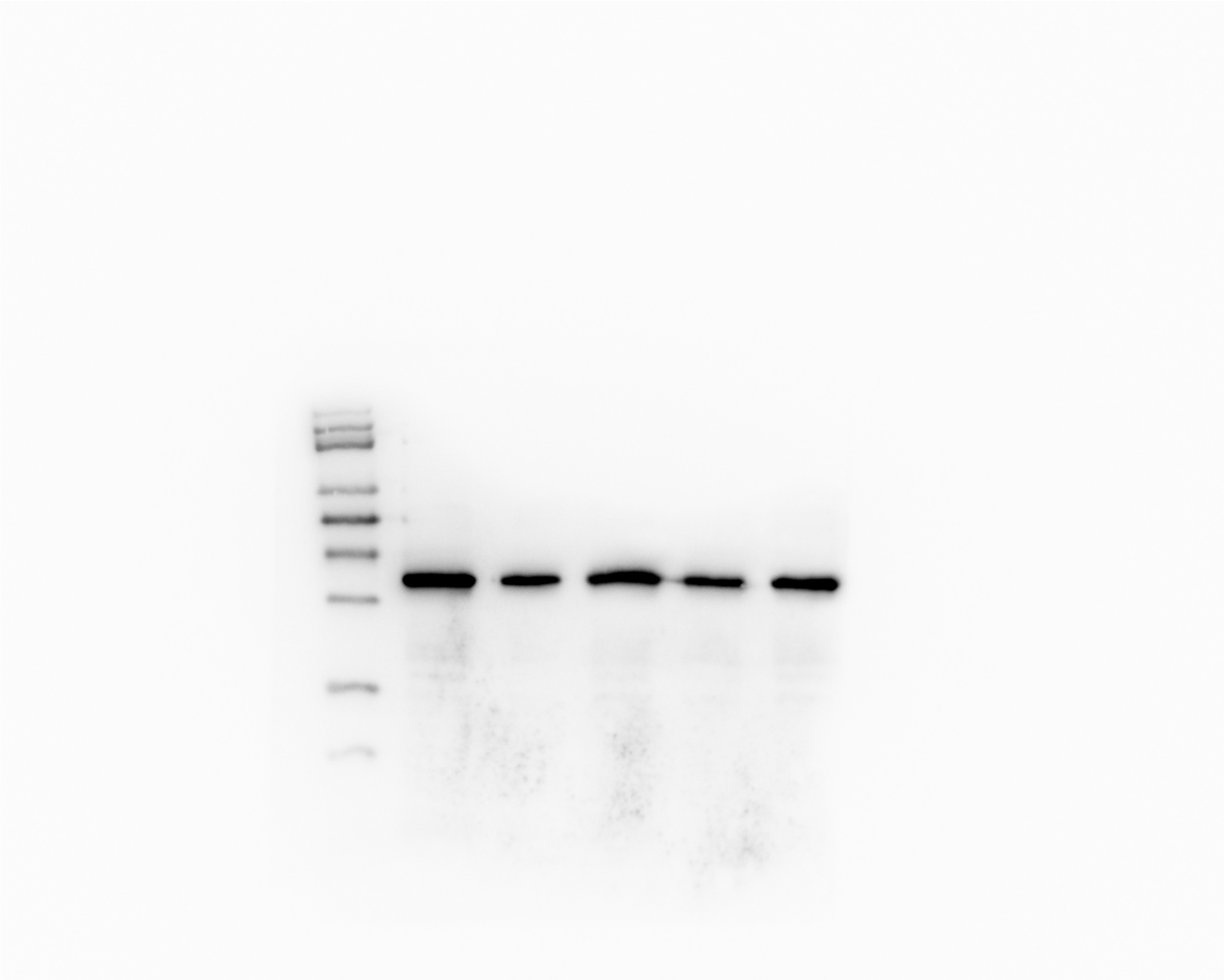

P-ERK-2

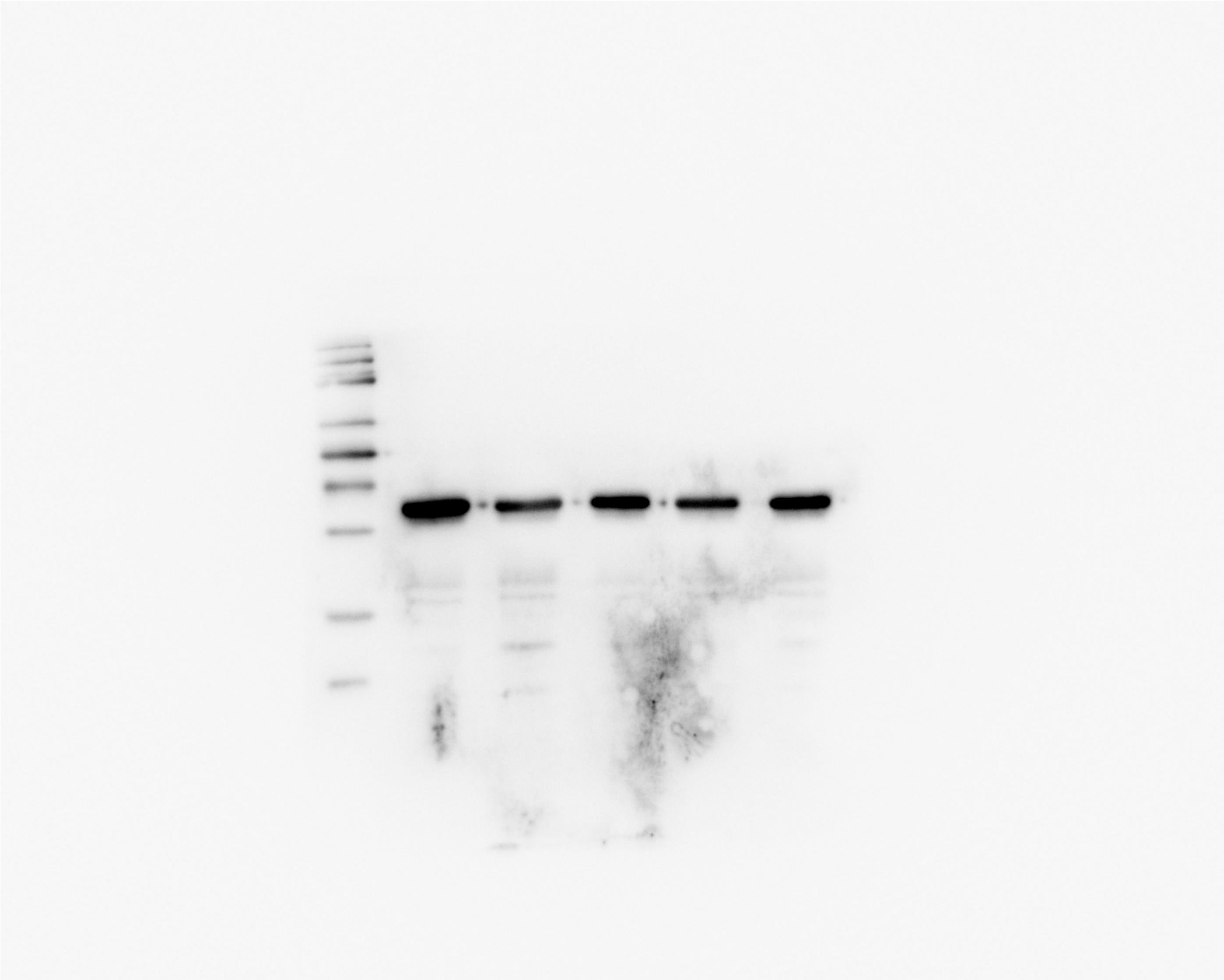

P-ERK-3

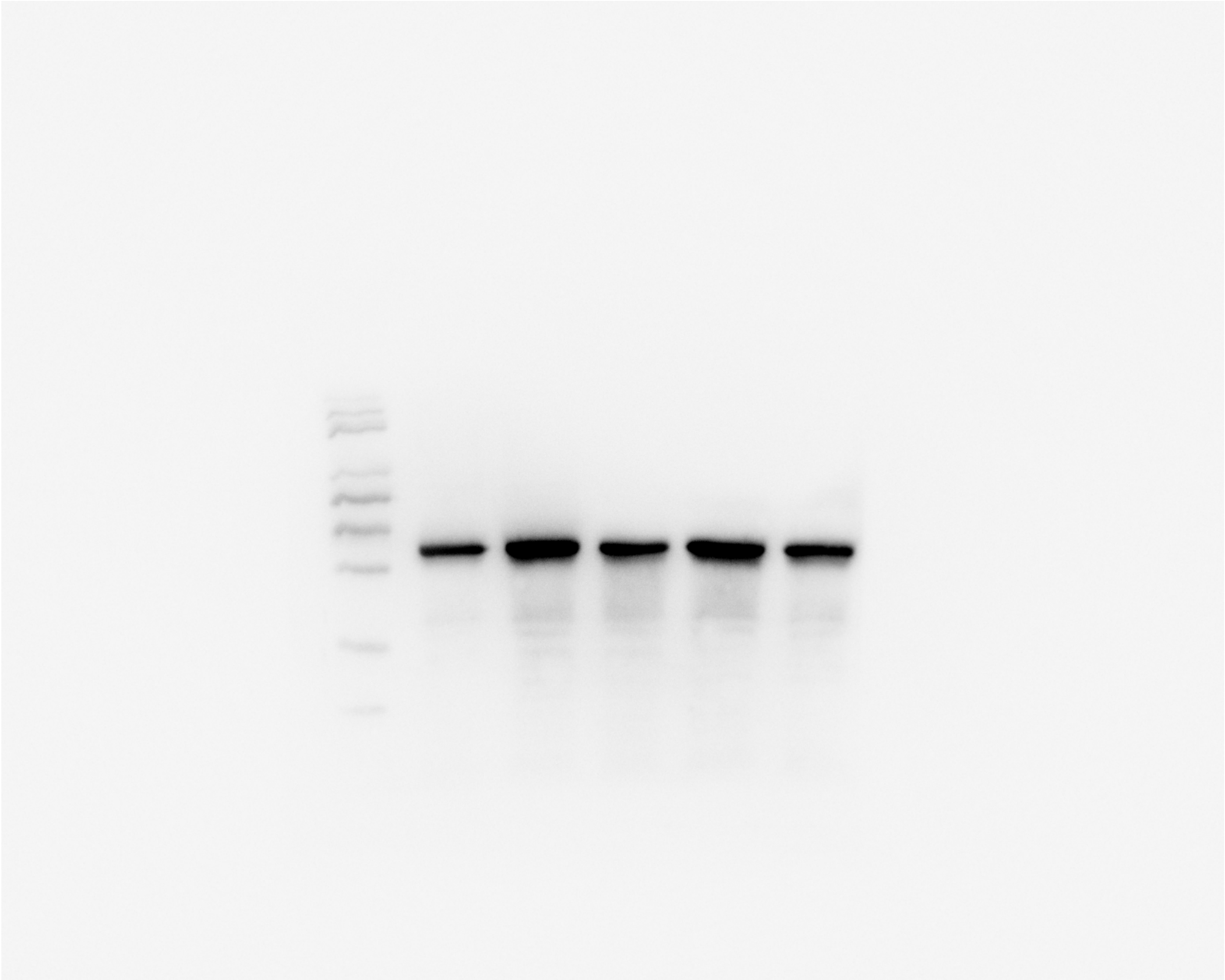

SRPINE1-1

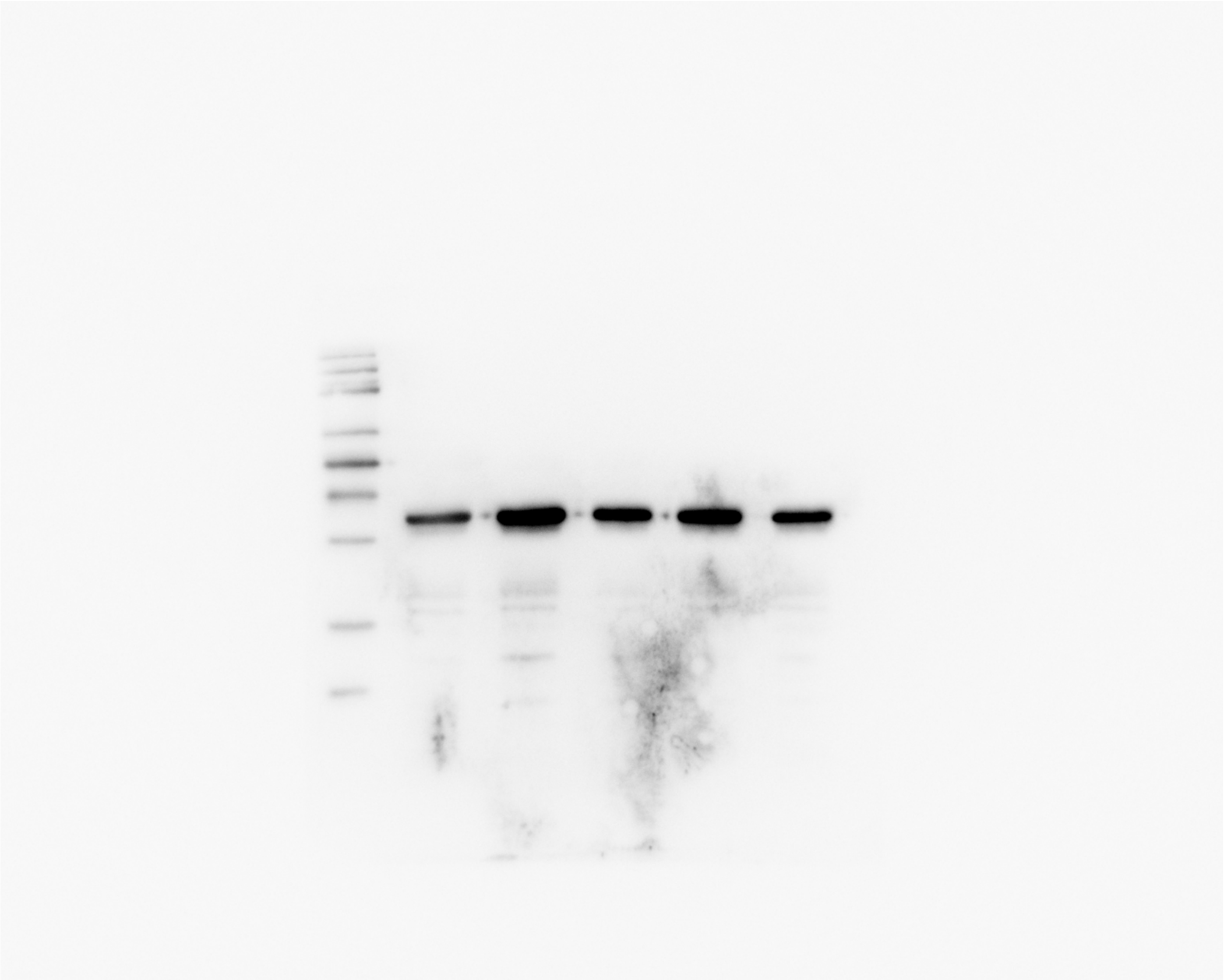

SRPINE1-2

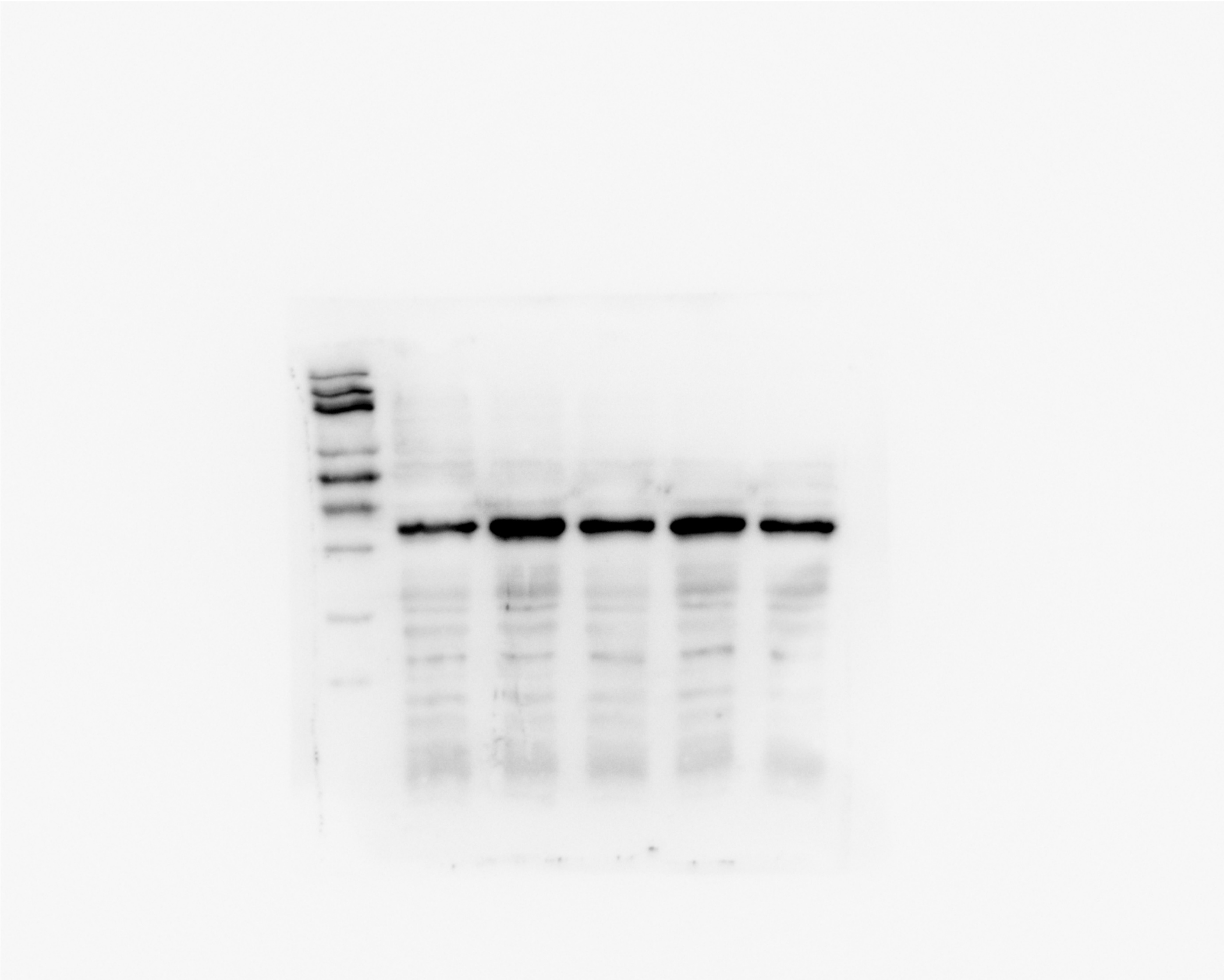

SRPINE1-3

Cropped blots:

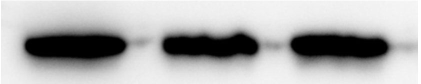

ERK-1

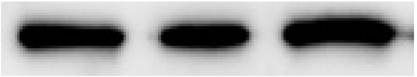

ERK-2

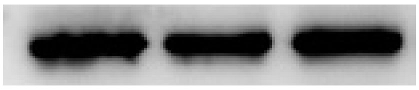

ERK-3

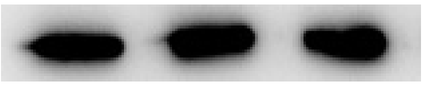

GAPDH-1

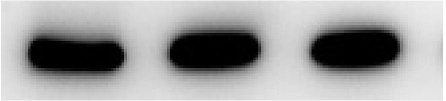

GAPDH-2

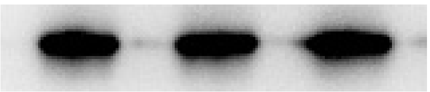

GAPDH-3

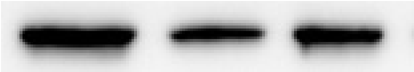

P-ERK-1

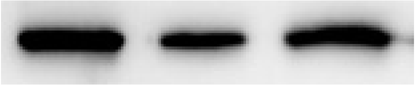

P-ERK-2

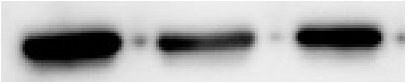

P-ERK-3

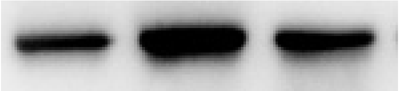

SRPINE1-1

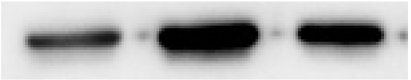

SRPINE1-2

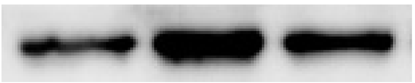

SRPINE1-3

Figure 4 F

Uncropped Blots:

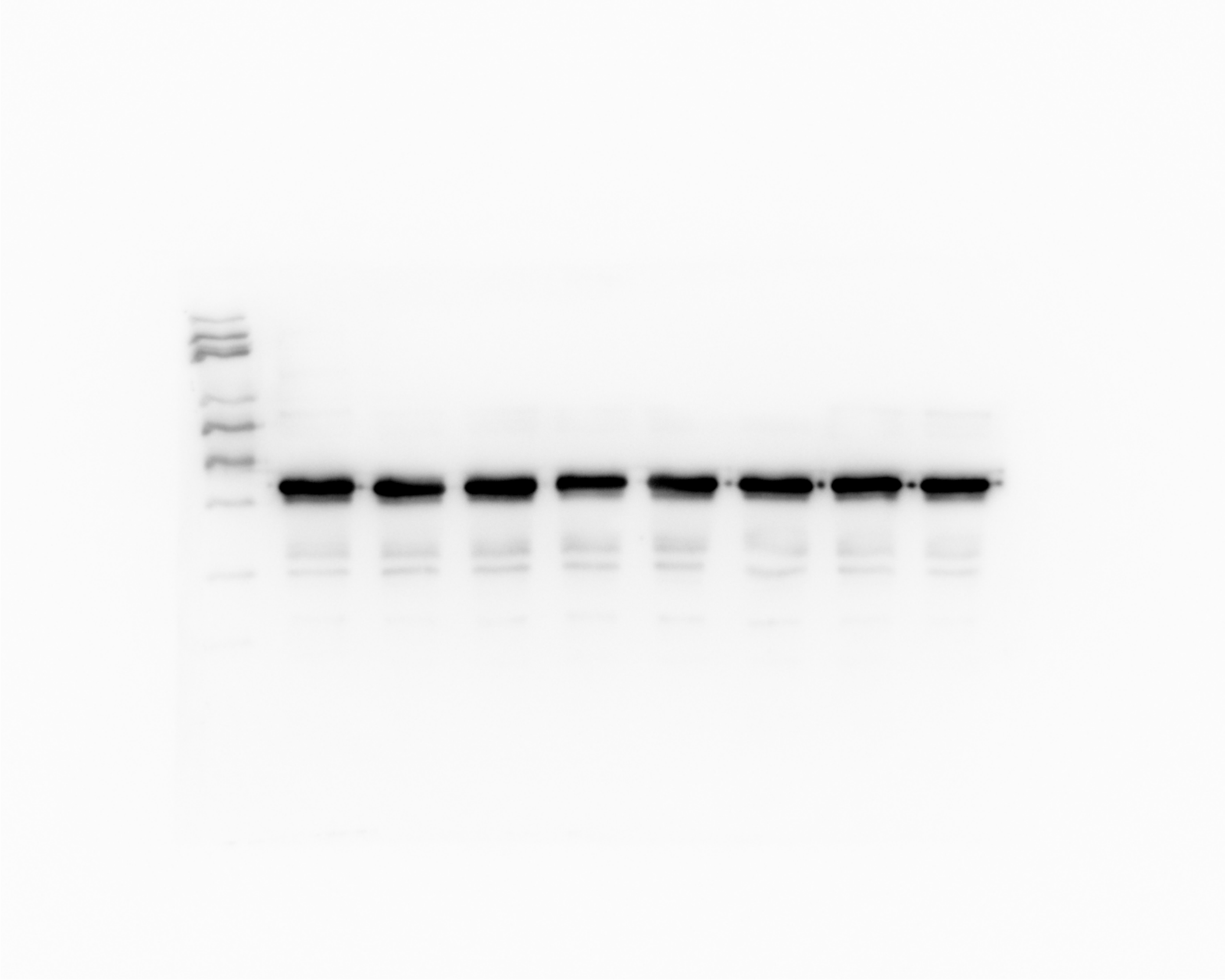

ERK-1

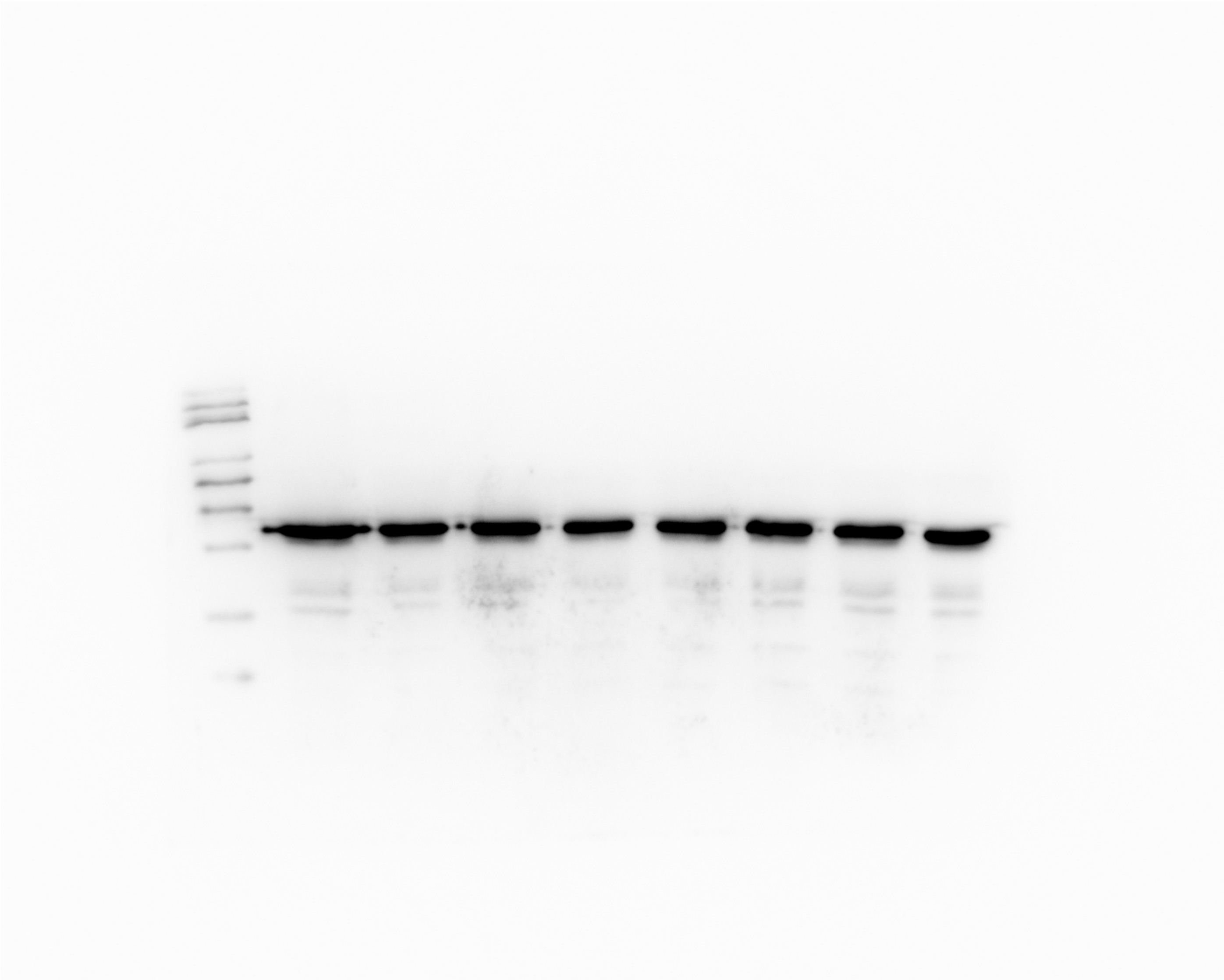

ERK-2

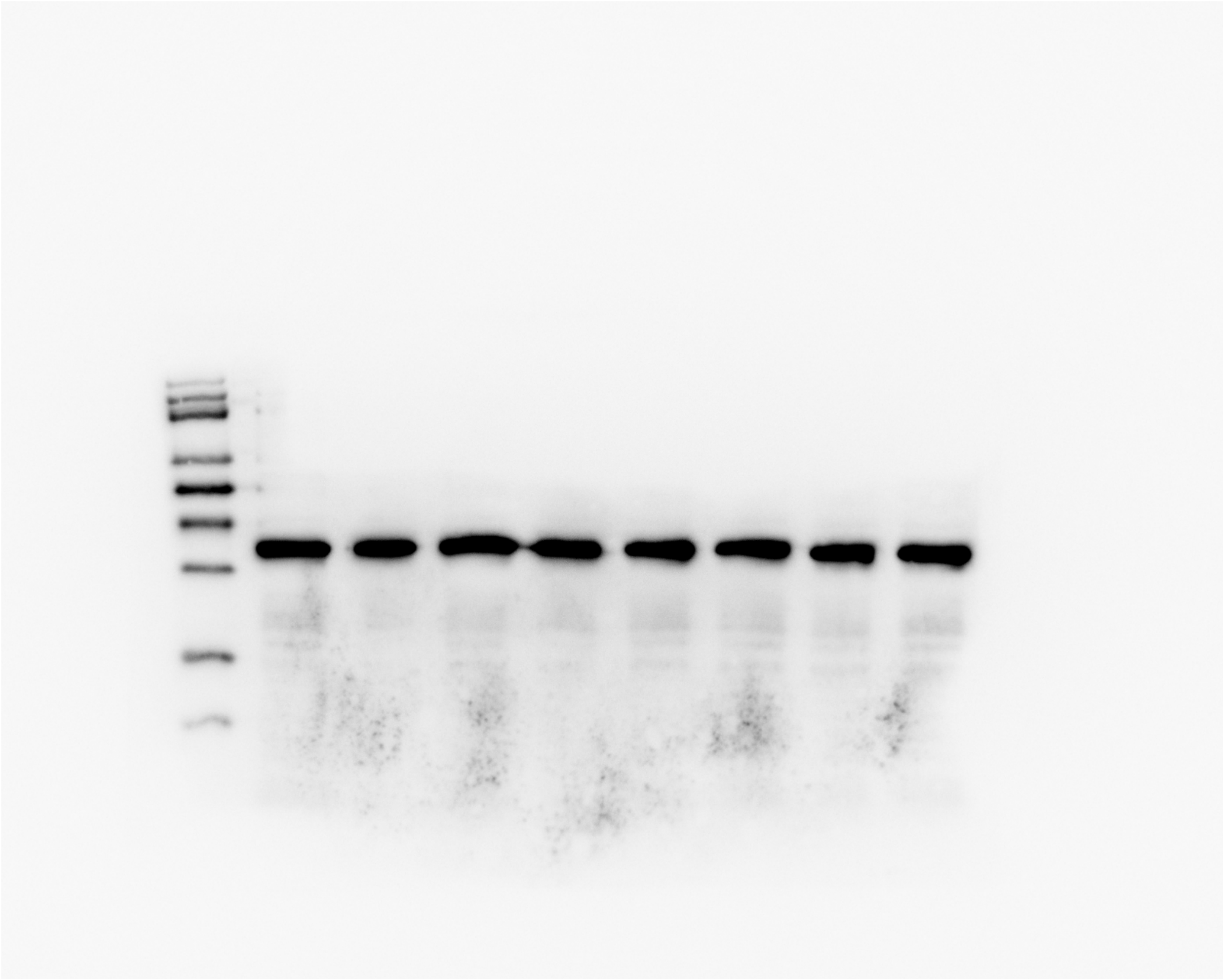

ERK-3

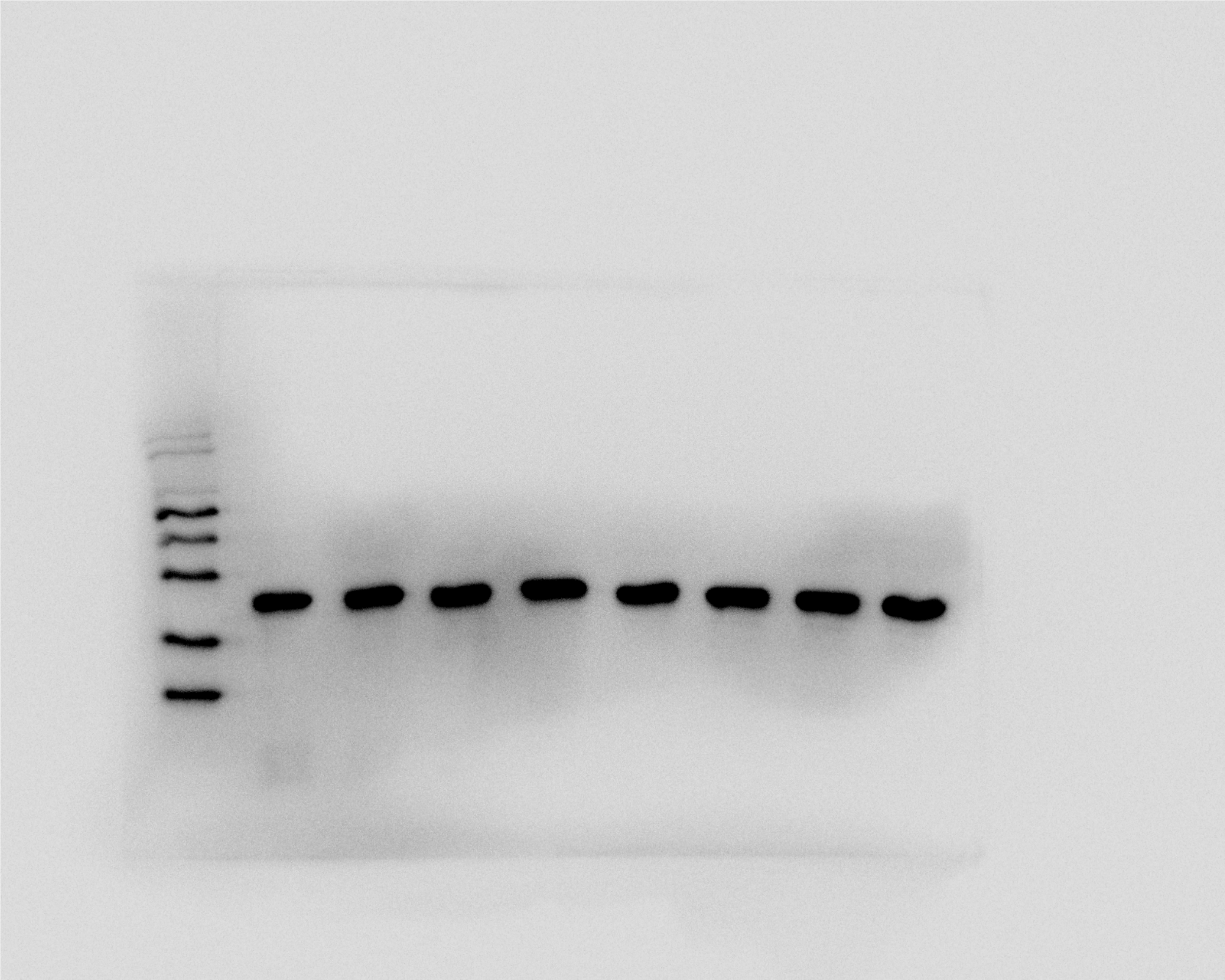

GAPDH-1

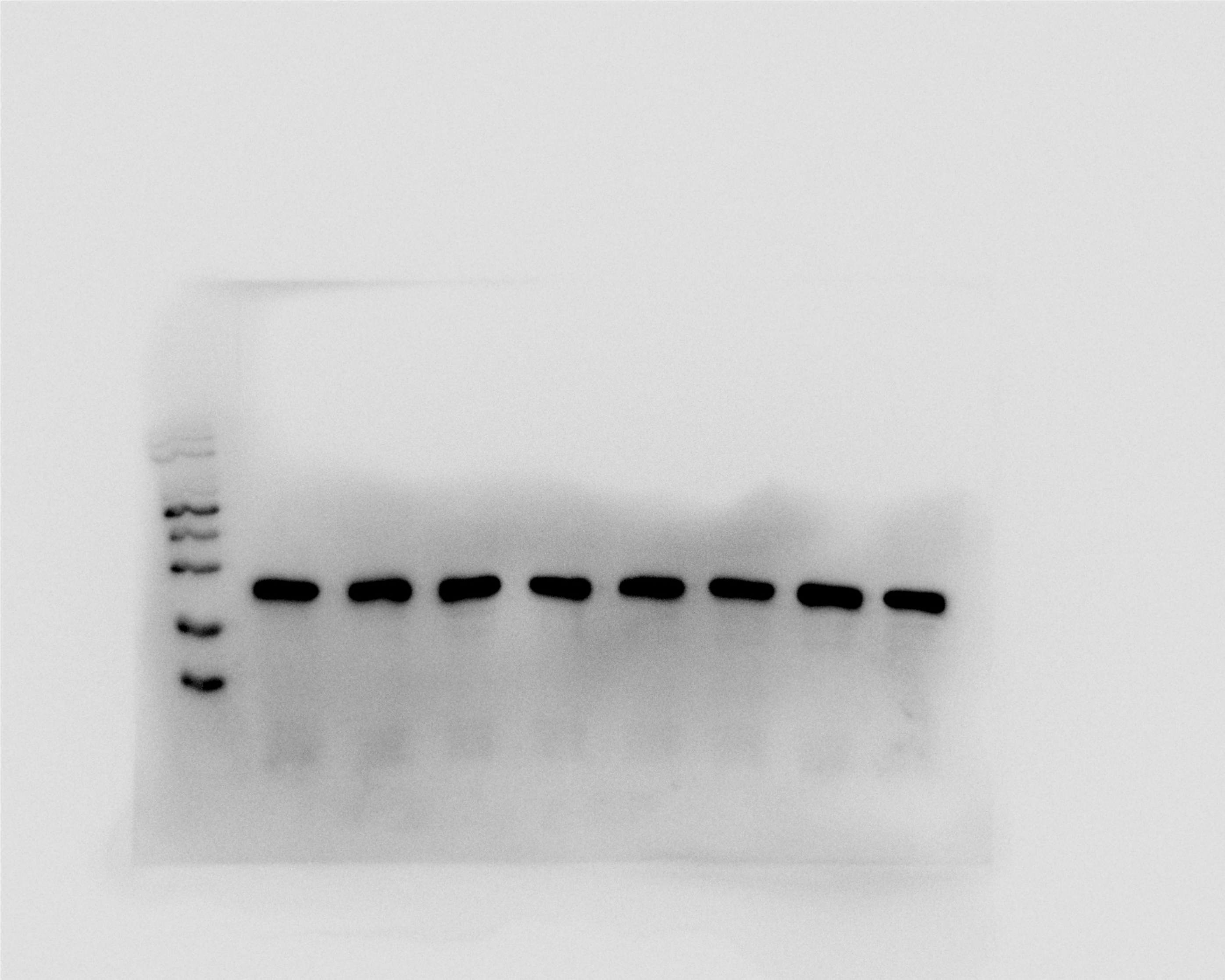

GAPDH-2

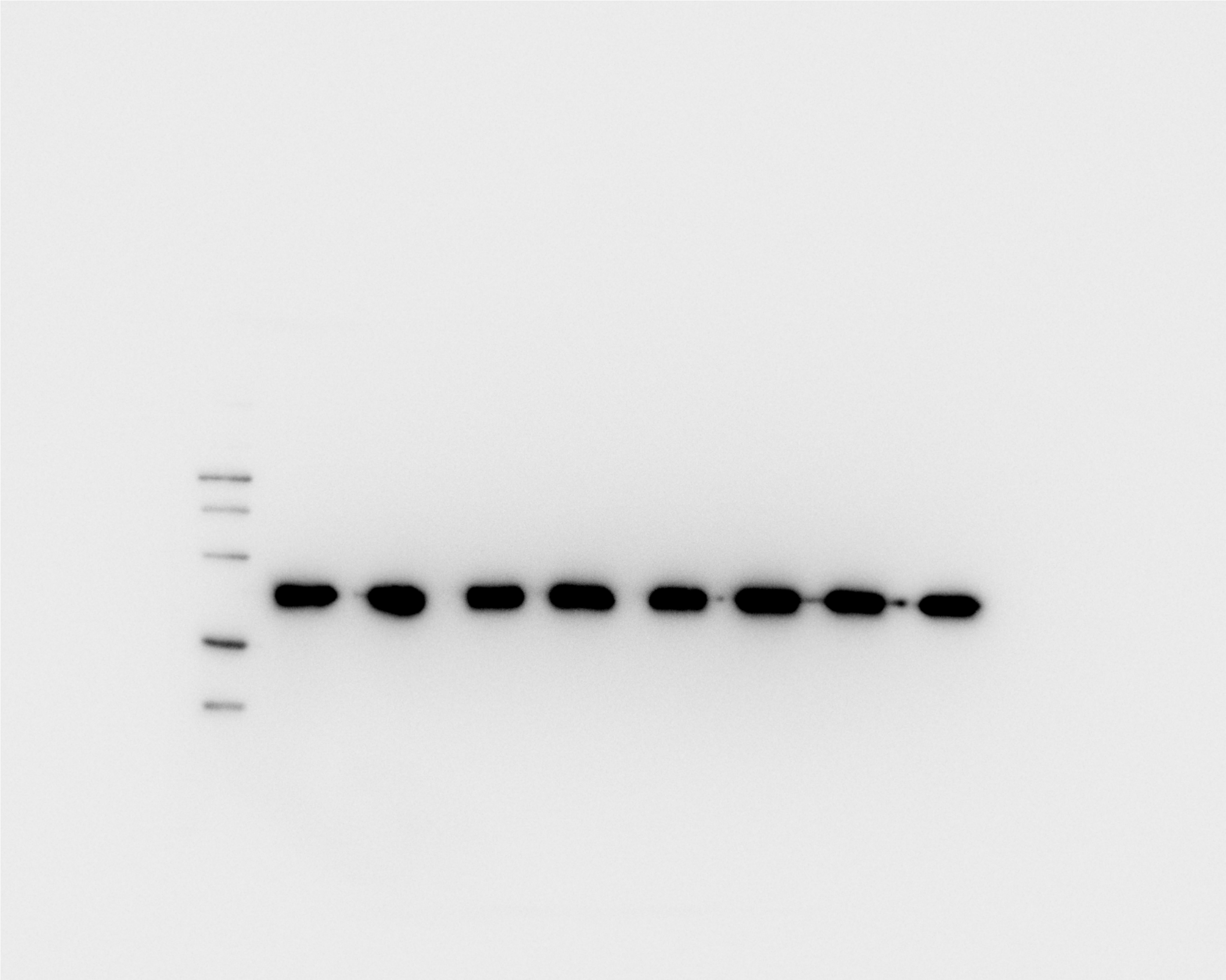

GAPDH-3

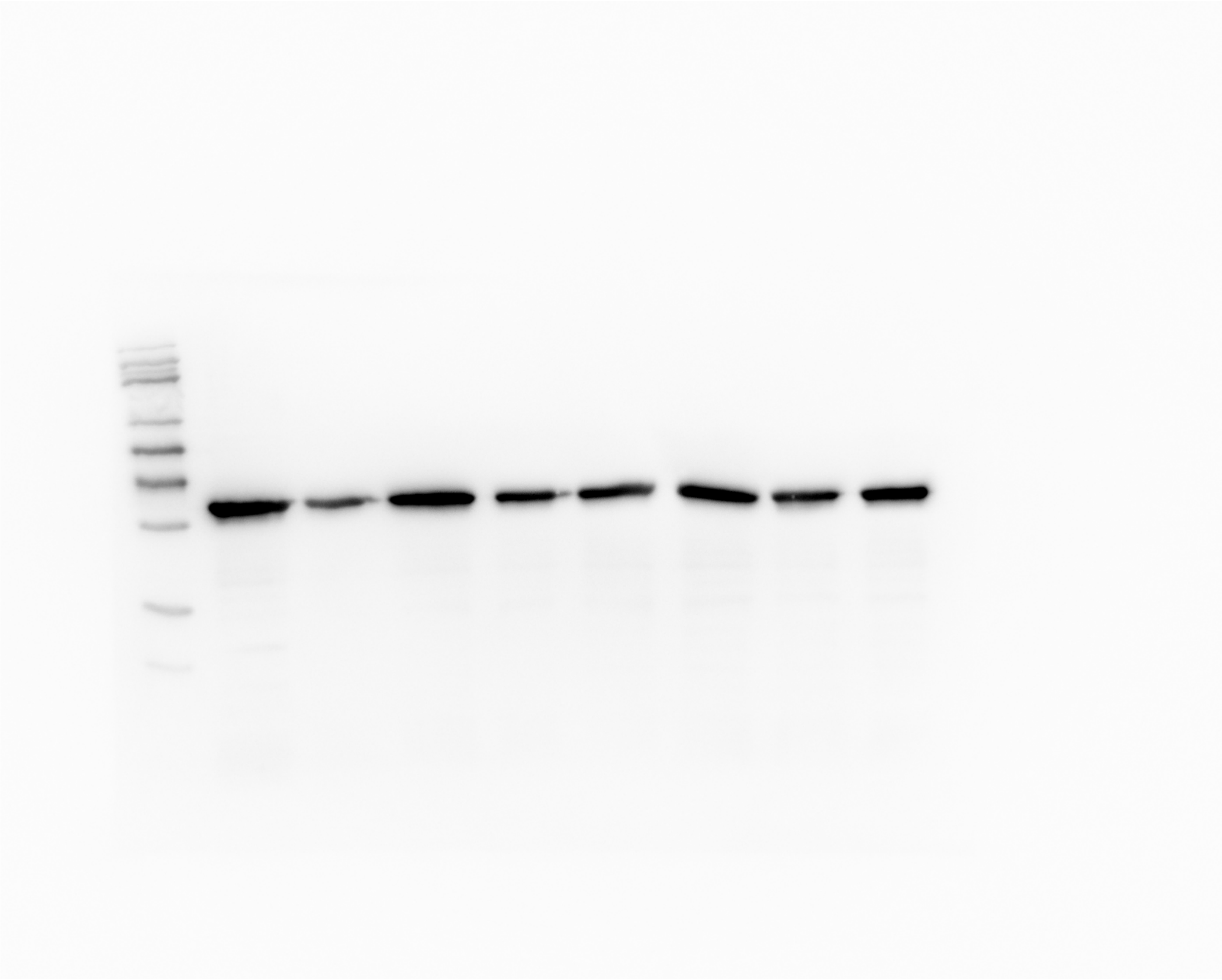

P-ERK-1

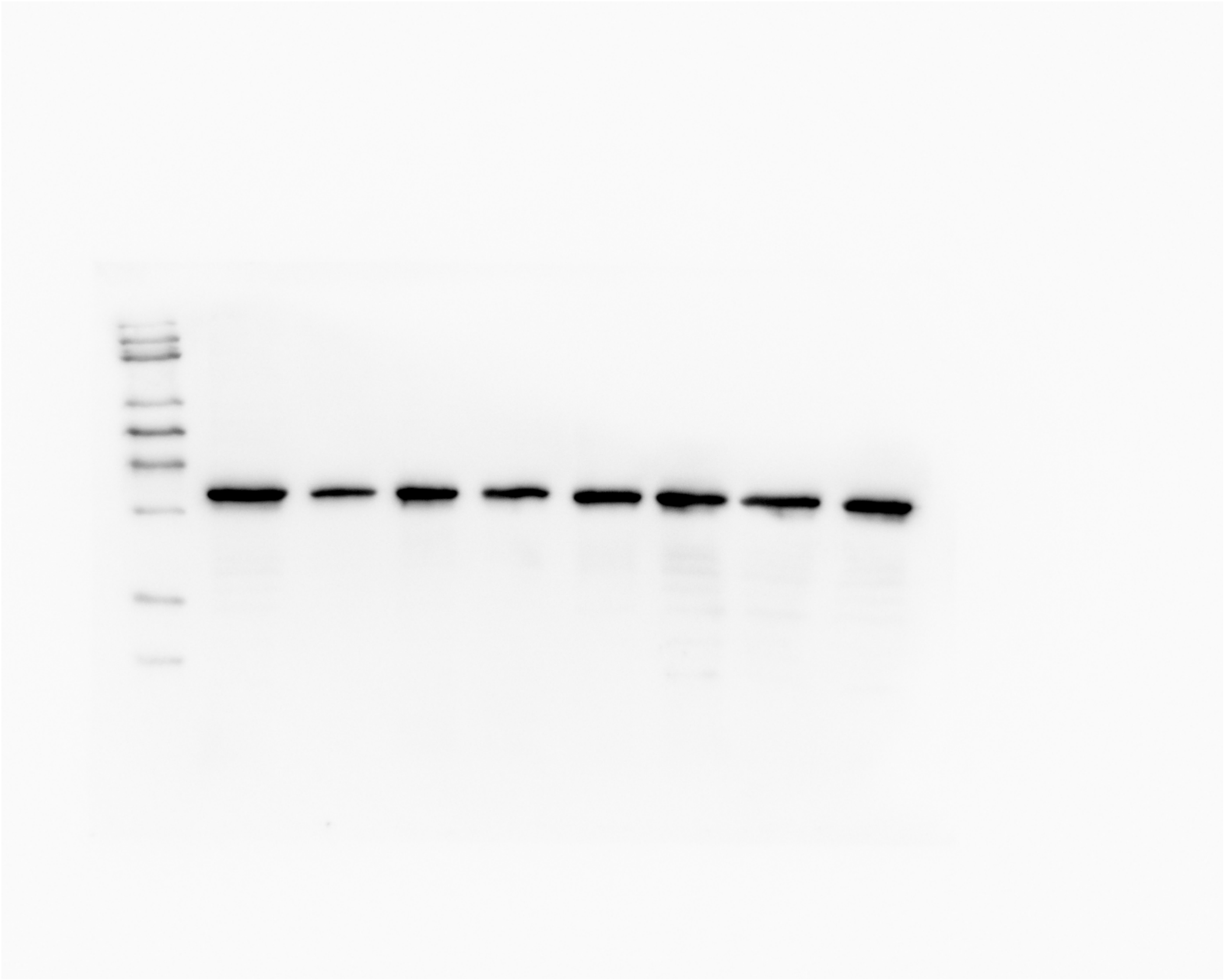

P-ERK-2

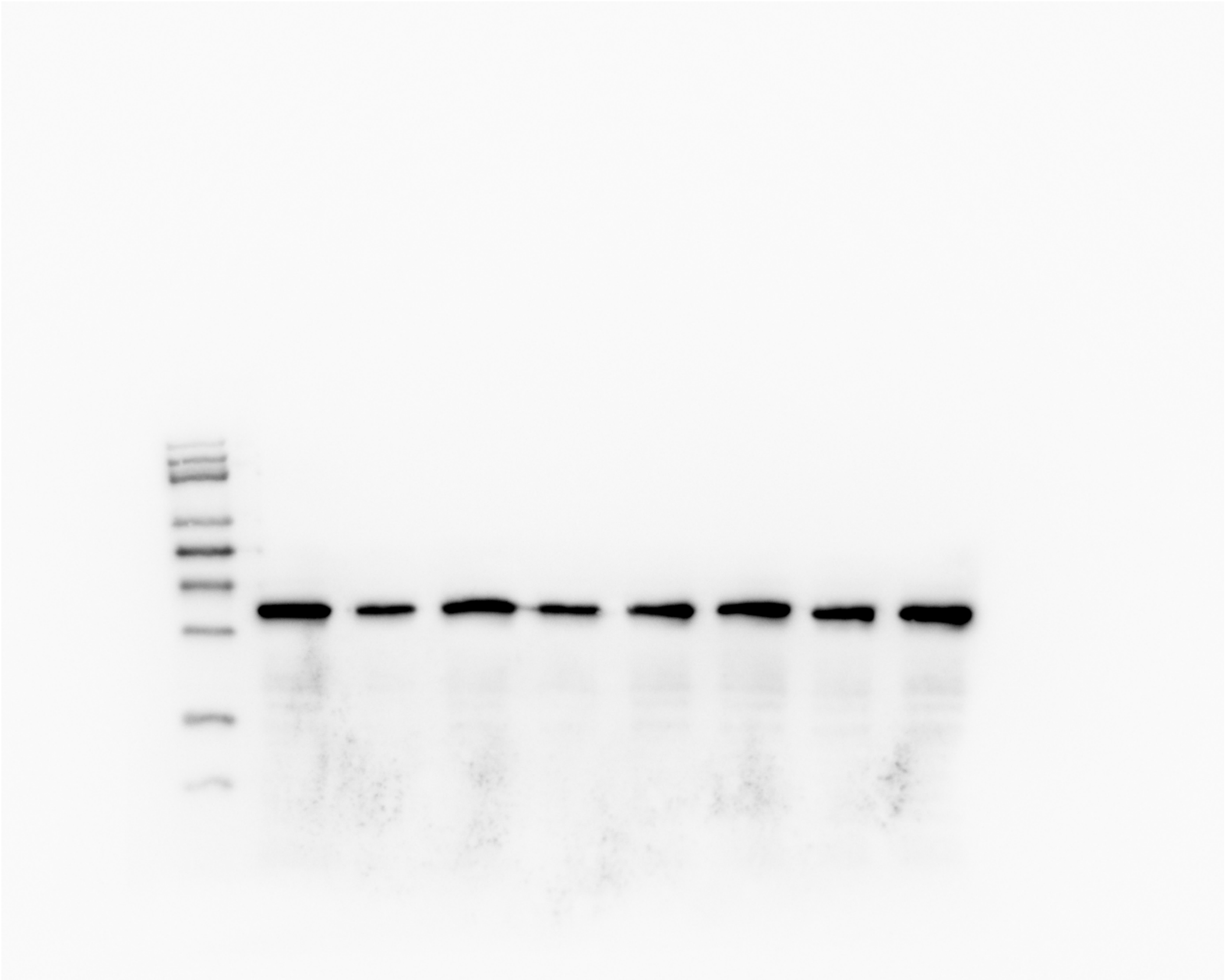

P-ERK-3

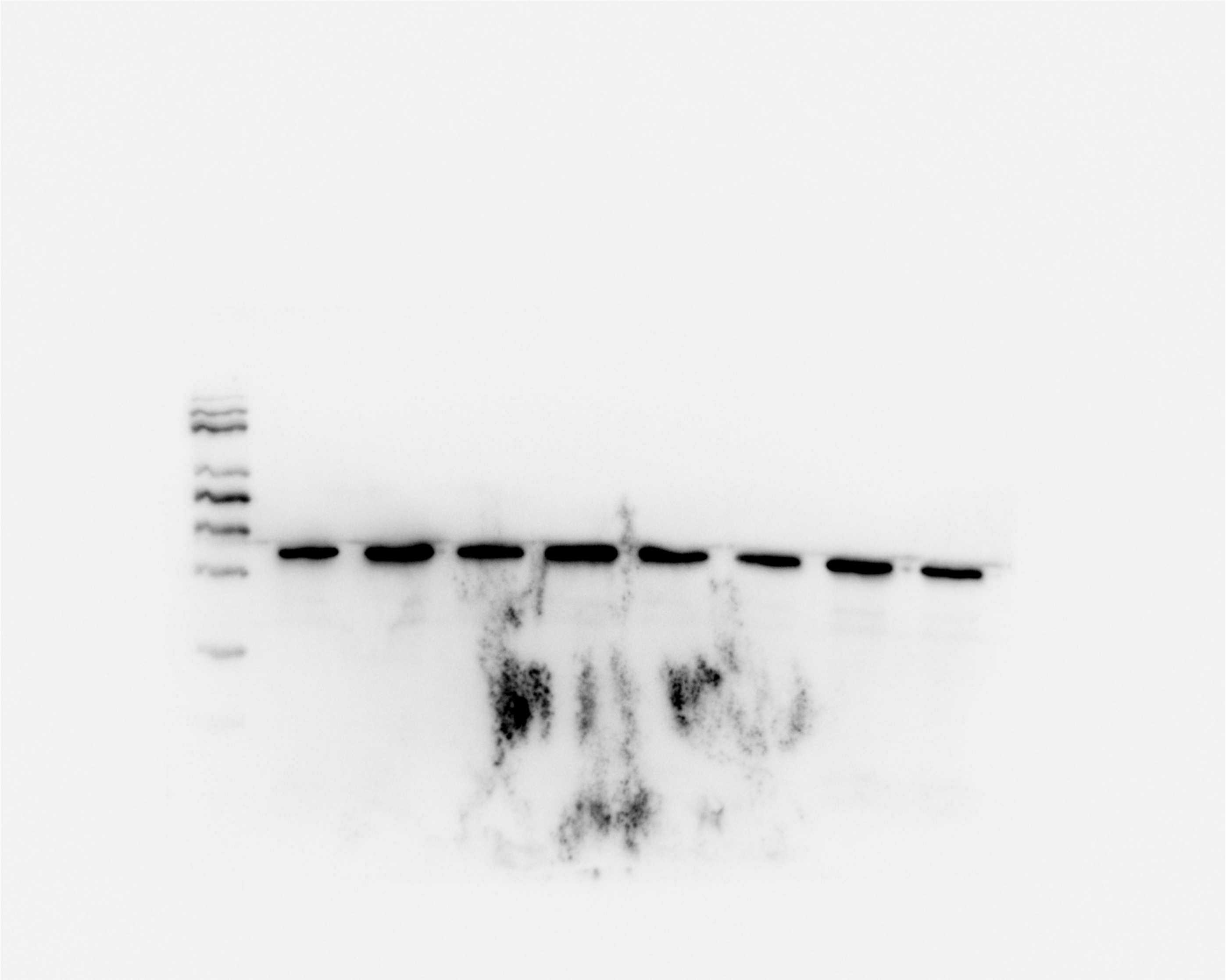

SERPINE1-1

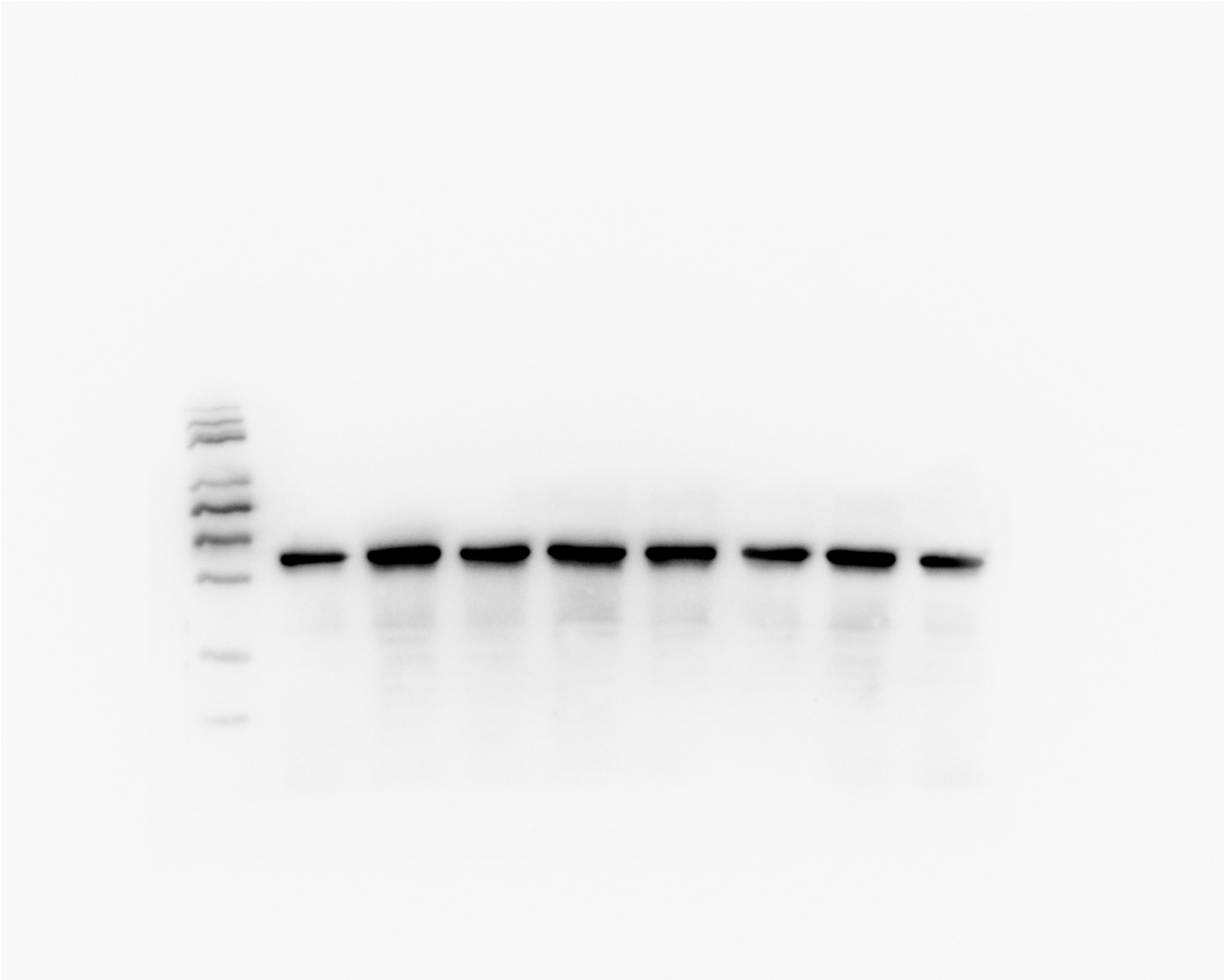

SERPINE1-2

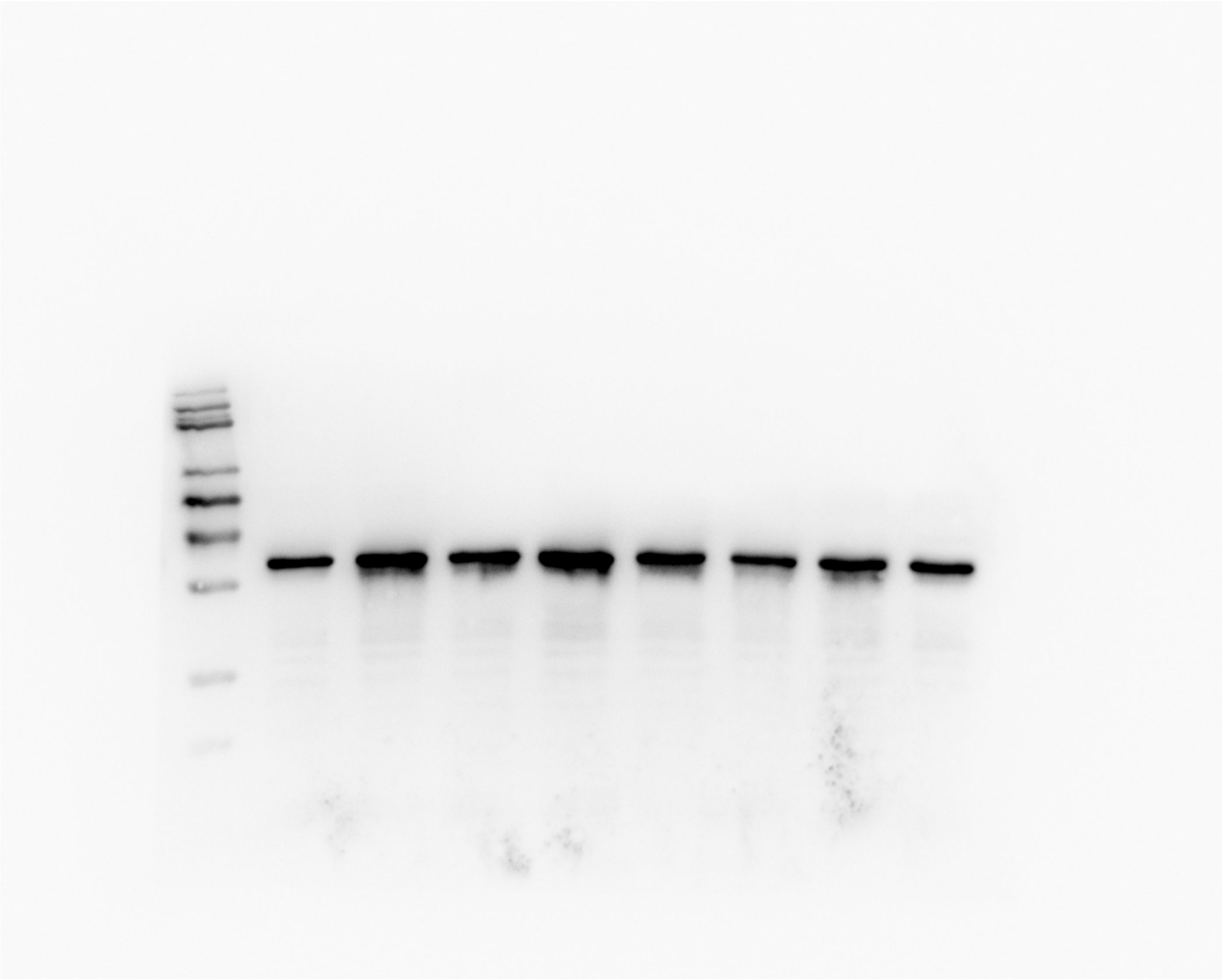

SERPINE1-3

Cropped blots:

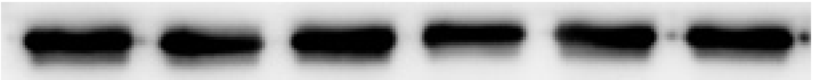

ERK-1

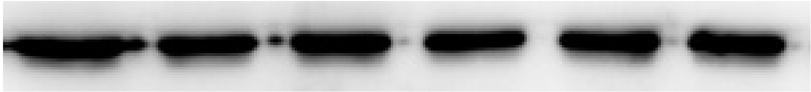

ERK-2

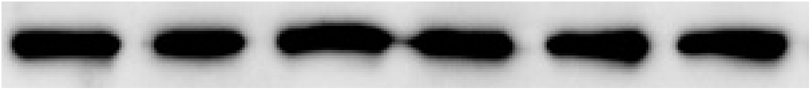

ERK-3

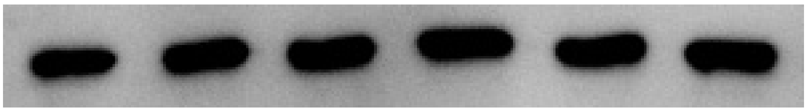

GAPDH-1

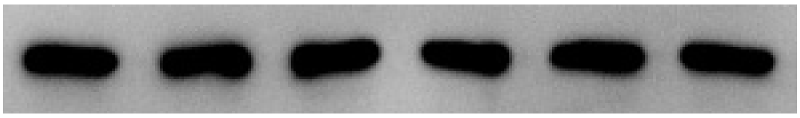

GAPDH-2

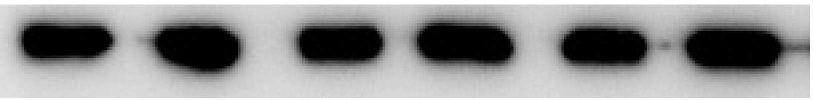

GAPDH-3

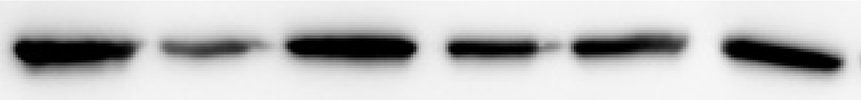

P-ERK-1

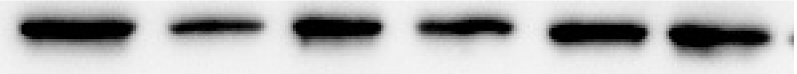

P-ERK-2

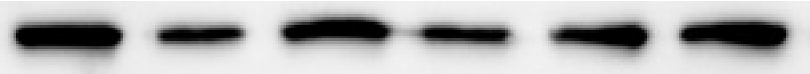

P-ERK-3

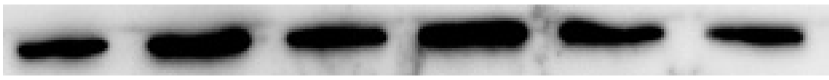

SERPINE1-1

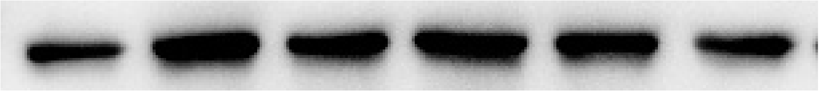

SERPINE1-2

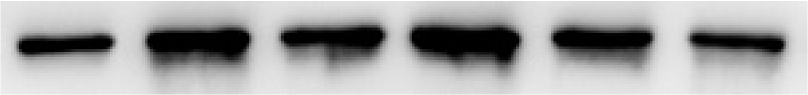

SERPINE1-3
